# Supplementary figures and images for: The importance of effect sizes when comparing cycle threshold values of SARS-CoV-2 variants
Source: PLoS One. 2022 Jul 21;17(7):e0271808. doi: 10.1371/journal.pone.0271808 (PMC9302753; doi:10.1371/journal.pone.0271808)

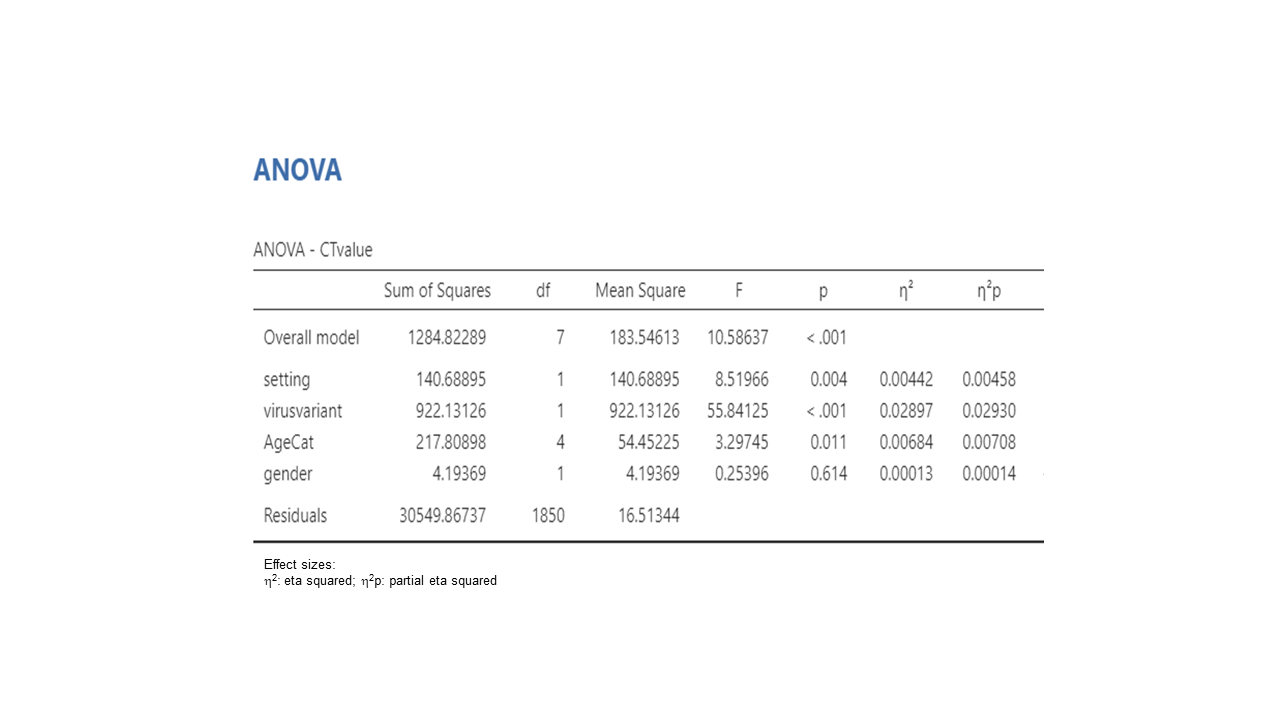

Supplement: S1 Table — (PNG) [file pone.0271808.s001.png]
