# Supplementary material for: The importance of effect sizes when comparing cycle threshold values of SARS-CoV-2 variants
Source: PLoS One. 2022 Jul 21;17(7):e0271808. doi: 10.1371/journal.pone.0271808 (PMC9302753; doi:10.1371/journal.pone.0271808)
Supplement: S1 Dataset — (PDF) [file pone.0271808.s002.pdf]

| CW | Gender f=1 m=2 | Age | CT value | PCR instrument Cobas=1 | Days since start of symptoms | Out-patient=1 In-patient=2 | 0=no mutation 1=british mutation 2=south african mutation |
|---|---|---|---|---|---|---|---|
| 3 | 1 | 55 | 21 | 1 | | 1 | 0 |
| 3 | 2 | 39 | 21 | 1 | | 1 | 0 |
| 3 | 2 | 8 | 31 | 1 | | 1 | 0 |
| 3 | 1 | 45 | 25 | 1 | | 1 | 0 |
| 3 | 2 | 55 | 28 | 1 | | 1 | 0 |
| 3 | 2 | 58 | 32 | 1 | | 1 | 0 |
| 3 | 1 | 100 | 22 | 1 | | 1 | 0 |
| 3 | 1 | 56 | 25 | 1 | | 1 | 0 |
| 3 | 1 | 15 | 28 | 1 | | 1 | 0 |
| 3 | 1 | 55 | 25 | 1 | | 1 | 0 |
| 3 | 1 | 23 | 31 | 1 | | 1 | 0 |
| 3 | 1 | 54 | 34 | 1 | | 1 | 0 |
| 3 | 2 | 47 | 20 | 1 | | 1 | 1 |
| 3 | 1 | 39 | 31 | 1 | | 1 | 0 |
| 3 | 2 | 59 | 16 | 1 | | 1 | 0 |
| 3 | 2 | 2 | 19 | 1 | | 1 | 0 |
| 3 | 1 | 47 | 21 | 1 | | 1 | 0 |
| 3 | 1 | 97 | 29 | 1 | | 1 | 0 |
| 3 | 2 | 23 | 33 | 1 | | 1 | 0 |
| 3 | 1 | 26 | 33 | 1 | | 1 | 0 |
| 3 | 2 | 56 | 22 | 1 | | 1 | 0 |
| 3 | 1 | 30 | 24 | 1 | | 1 | 0 |
| 3 | 2 | 51 | 27 | 1 | | 1 | 0 |
| 3 | 1 | 24 | 31 | 1 | | 1 | 0 |
| 3 | 1 | 36 | 23 | 1 | | 1 | 0 |
| 3 | 2 | 50 | 21 | 1 | | 1 | 0 |
| 3 | 1 | 23 | 25 | 1 | | 1 | 0 |
| 3 | 2 | 9 | 30 | 1 | | 1 | 0 |
| 3 | 2 | 27 | 21 | 1 | | 1 | 0 |
| 3 | 1 | 42 | 31 | 1 | | 1 | 0 |
| 3 | 2 | 59 | 34 | 1 | | 1 | 0 |
| 3 | 1 | 38 | 27 | 1 | | 1 | 0 |
| 3 | 2 | 61 | 32 | 1 | | 1 | 1 |
| 3 | 1 | 30 | 23 | 1 | | 1 | 0 |
| 3 | 1 | 48 | 24 | 1 | | 1 | 0 |
| 3 | 2 | 18 | 30 | 1 | | 1 | 0 |
| 3 | 2 | 19 | 35 | 1 | | 1 | 0 |
| 3 | 1 | 71 | 24 | 1 | | 1 | 0 |
| 3 | 2 | 36 | 29 | 1 | | 1 | 0 |
| 3 | 1 | 69 | 22 | 1 | | 1 | 1 |

| | | | | | | |
|---|---|---|---|---|---|---|
| 3 | 1 | 80 | 26 | 1 | 1 | 0 |
| 3 | 1 | 27 | 25 | 1 | 1 | 0 |
| 3 | 2 | 37 | 26 | 1 | 1 | 0 |
| 3 | 1 | 61 | 30 | 1 | 1 | 0 |
| 3 | 1 | 59 | 32 | 1 | 1 | 0 |
| 3 | 2 | 21 | 24 | 1 | 1 | 0 |
| 3 | 2 | 50 | 24 | 1 | 1 | 0 |
| 3 | 1 | 48 | 24 | 1 | 1 | 0 |
| 3 | 1 | 39 | 27 | 1 | 1 | 0 |
| 3 | 2 | 29 | 30 | 1 | 1 | 0 |
| 3 | 1 | 33 | 30 | 1 | 1 | 0 |
| 3 | 1 | 66 | 31 | 1 | 2 | 0 |
| 3 | 1 | 52 | 32 | 1 | 2 | 0 |
| 3 | 2 | 90 | 31 | 1 | 1 | 0 |
| 3 | 2 | 82 | 32 | 1 | 2 | 0 |
| 3 | 2 | 69 | 35 | 1 | 2 | 0 |
| 3 | 2 | 60 | 21 | 1 | 1 | 0 |
| 3 | 2 | 33 | 21 | 1 | 1 | 0 |
| 3 | 1 | 33 | 29 | 1 | 1 | 0 |
| 3 | 1 | 49 | 25 | 1 | 1 | 0 |
| 3 | 1 | 86 | 21 | 1 | 1 | 0 |
| 3 | 1 | 34 | 22 | 1 | 1 | 0 |
| 3 | 2 | 43 | 26 | 1 | 1 | 0 |
| 3 | 2 | 41 | 31 | 1 | 1 | 0 |
| 3 | 2 | 28 | 32 | 1 | 1 | 0 |
| 3 | 2 | 78 | 33 | 1 | 1 | 0 |
| 3 | 2 | 20 | 27 | 1 | 1 | 0 |
| 3 | 1 | 41 | 32 | 1 | 1 | 0 |
| 3 | 1 | 52 | 24 | 1 | 1 | 0 |
| 3 | 1 | 58 | 24 | 1 | 1 | 0 |
| 3 | 2 | 34 | 33 | 1 | 1 | 0 |
| 3 | 1 | 30 | 33 | 1 | 1 | 0 |
| 3 | 1 | 52 | 20 | 1 | 1 | 0 |
| 3 | 1 | 47 | 22 | 1 | 1 | 0 |
| 3 | 2 | 59 | 23 | 1 | 1 | 0 |
| 3 | 1 | 64 | 26 | 1 | 1 | 0 |
| 3 | 1 | 76 | 31 | 1 | 1 | 0 |
| 3 | 1 | 1 | 34 | 1 | 1 | 0 |
| 3 | 2 | 39 | 34 | 1 | 1 | 1 |
| 3 | 1 | 81 | 32 | 1 | 1 | 0 |
| 3 | 2 | 61 | 31 | 1 | 1 | 0 |
| 3 | 2 | 74 | 32 | 1 | 1 | 0 |
| 3 | 2 | 73 | 22 | 1 | 1 | 0 |
| 3 | 1 | 41 | 32 | 1 | 1 | 0 |
| 3 | 2 | 61 | 25 | 1 | 1 | 0 |
| 3 | 1 | 64 | 34 | 1 | 1 | 0 |
| 3 | 2 | 52 | 25 | 1 | 1 | 0 |
| 3 | 2 | 39 | 26 | 1 | 1 | 0 |
| 3 | 2 | 67 | 30 | 1 | 1 | 0 |
| 3 | 2 | 66 | 26 | 1 | 1 | 0 |

| | | | | | | |
|---|---|---|---|---|---|---|
| 3 | 1 | 42 | 27 | 1 | 1 | 0 |
| 3 | 1 | 70 | 27 | 1 | 1 | 0 |
| 3 | 2 | 18 | 33 | 1 | 1 | 0 |
| 3 | 2 | 37 | 34 | 1 | 1 | 0 |
| 3 | 2 | 70 | 33 | 1 | 1 | 0 |
| 3 | 2 | 54 | 26 | 1 | 1 | 0 |
| 3 | 1 | 51 | 31 | 1 | 1 | 0 |
| 3 | 1 | 20 | 35 | 1 | 1 | 0 |
| 3 | 2 | 44 | 25 | 1 | 1 | 1 |
| 3 | 1 | 97 | 27 | 1 | 1 | 0 |
| 3 | 1 | 99 | 27 | 1 | 1 | 0 |
| 3 | 1 | 14 | 25 | 1 | 1 | 0 |
| 3 | 2 | 58 | 33 | 1 | 1 | 0 |
| 3 | 2 | 84 | 15 | 1 | 2 | 0 |
| 4 | 2 | 46 | 21 | 1 | 2 | 0 |
| 4 | 2 | 59 | 22 | 1 | 1 | 0 |
| 4 | 1 | 51 | 27 | 1 | 1 | 0 |
| 4 | 2 | 11 | 34 | 1 | 1 | 0 |
| 4 | 2 | 86 | 28 | 1 | 2 | 0 |
| 4 | 1 | 46 | 20 | 1 | 1 | 0 |
| 4 | 1 | 24 | 24 | 1 | 1 | 0 |
| 4 | 2 | 37 | 31 | 1 | 1 | 0 |
| 4 | 1 | 46 | 21 | 1 | 1 | 0 |
| 4 | 1 | 71 | 21 | 1 | 1 | 0 |
| 4 | 1 | 52 | 21 | 1 | 1 | 0 |
| 4 | 1 | 51 | 29 | 1 | 1 | 0 |
| 4 | 1 | 38 | 23 | 1 | 1 | 0 |
| 4 | 1 | 44 | 24 | 1 | 1 | 0 |
| 4 | 1 | 39 | 24 | 1 | 1 | 0 |
| 4 | 1 | 85 | 25 | 1 | 2 | 0 |
| 4 | 1 | 22 | 28 | 1 | 1 | 0 |
| 4 | 2 | 28 | 19 | 1 | 1 | 0 |
| 4 | 1 | 38 | 20 | 1 | 1 | 0 |
| 4 | 2 | 34 | 21 | 1 | 1 | 0 |
| 4 | 2 | 62 | 23 | 1 | 1 | 0 |
| 4 | 2 | 47 | 24 | 1 | 1 | 0 |
| 4 | 1 | 43 | 25 | 1 | 1 | 0 |
| 4 | 2 | 83 | 27 | 1 | 1 | 0 |
| 4 | 1 | 60 | 29 | 1 | 1 | 0 |
| 4 | 1 | 41 | 30 | 1 | 1 | 0 |
| 4 | 2 | 64 | 23 | 1 | 1 | 1 |
| 4 | 1 | 37 | 31 | 1 | 1 | 0 |
| 4 | 1 | 39 | 33 | 1 | 1 | 0 |
| 4 | 1 | 39 | 28 | 1 | 1 | 0 |
| 4 | 1 | 58 | 32 | 1 | 1 | 0 |
| 4 | 1 | 62 | 33 | 1 | 1 | 0 |
| 4 | 2 | 73 | 21 | 1 | 1 | 0 |
| 4 | 1 | 41 | 27 | 1 | 1 | 0 |
| 4 | 2 | 43 | 32 | 1 | 1 | 0 |
| 4 | 1 | 32 | 32 | 1 | 1 | 0 |

| | | | | | | |
|---|---|---|---|---|---|---|
| 4 | 1 | 71 | 31 | 1 | 1 | 0 |
| 4 | 2 | 4 | 22 | 1 | 1 | 0 |
| 4 | 2 | 53 | 23 | 1 | 1 | 0 |
| 4 | 2 | 22 | 27 | 1 | 1 | 0 |
| 4 | 2 | 49 | 28 | 1 | 1 | 0 |
| 4 | 1 | 66 | 29 | 1 | 2 | 0 |
| 4 | 1 | 66 | 34 | 1 | 2 | 0 |
| 4 | 1 | 81 | 29 | 1 | 2 | 0 |
| 4 | 2 | 52 | 27 | 1 | 1 | 0 |
| 4 | 2 | 64 | 26 | 1 | 1 | 0 |
| 4 | 1 | 36 | 25 | 1 | 1 | 0 |
| 4 | 1 | 47 | 33 | 1 | 1 | 0 |
| 4 | 1 | 21 | 21 | 1 | 1 | 0 |
| 4 | 1 | 77 | 34 | 1 | 1 | 0 |
| 4 | 1 | 21 | 21 | 1 | 1 | 0 |
| 4 | 2 | 54 | 30 | 1 | 1 | 0 |
| 4 | 1 | 51 | 20 | 1 | 1 | 0 |
| 4 | 1 | 86 | 21 | 1 | 1 | 1 |
| 4 | 1 | 93 | 22 | 1 | 1 | 1 |
| 4 | 2 | 61 | 33 | 1 | 1 | 1 |
| 4 | 2 | 37 | 25 | 1 | 1 | 0 |
| 4 | 2 | 37 | 31 | 1 | 1 | 0 |
| 4 | 2 | 52 | 26 | 1 | 1 | 0 |
| 4 | 1 | 52 | 32 | 1 | 1 | 0 |
| 4 | 1 | 51 | 22 | 1 | 1 | 0 |
| 4 | 1 | 1 | 32 | 1 | 1 | 0 |
| 4 | 2 | 42 | 23 | 1 | 1 | 0 |
| 4 | 2 | 42 | 24 | 1 | 1 | 0 |
| 4 | 2 | 62 | 31 | 1 | 1 | 0 |
| 4 | 1 | 28 | 22 | 1 | 1 | 0 |
| 4 | 1 | 55 | 28 | 1 | 1 | 0 |
| 4 | 2 | 64 | 32 | 1 | 1 | 0 |
| 4 | 1 | 63 | 34 | 1 | 1 | 0 |
| 4 | 1 | 28 | 24 | 1 | 1 | 0 |
| 4 | 2 | 26 | 32 | 1 | 1 | 0 |
| 4 | 1 | 72 | 32 | 1 | 1 | 0 |
| 4 | 2 | 74 | 33 | 1 | 1 | 0 |
| 4 | 2 | 39 | 33 | 1 | 1 | 0 |
| 4 | 1 | 21 | 19 | 1 | 1 | 0 |
| 4 | 1 | 51 | 23 | 1 | 1 | 0 |
| 4 | 1 | 60 | 32 | 1 | 1 | 0 |
| 4 | 1 | 32 | 25 | 1 | 1 | 0 |
| 4 | 2 | 38 | 29 | 1 | 1 | 0 |
| 4 | 1 | 52 | 22 | 1 | 1 | 0 |
| 4 | 1 | 17 | 27 | 1 | 1 | 0 |
| 4 | 1 | 25 | 27 | 1 | 1 | 0 |
| 4 | 1 | 29 | 27 | 1 | 1 | 1 |
| 4 | 1 | 41 | 18 | 1 | 1 | 0 |
| 4 | 1 | 43 | 27 | 1 | 1 | 0 |
| 4 | 1 | 24 | 28 | 1 | 1 | 0 |

| | | | | | | |
|---|---|---|---|---|---|---|
| 4 | 1 | 61 | 32 | 1 | 1 | 0 |
| 4 | 1 | 46 | 26 | 1 | 1 | 0 |
| 4 | 2 | 59 | 28 | 1 | 1 | 0 |
| 4 | 2 | 56 | 28 | 1 | 1 | 0 |
| 4 | 2 | 77 | 25 | 1 | 1 | 0 |
| 4 | 1 | 97 | 23 | 1 | 1 | 0 |
| 4 | 2 | 25 | 33 | 1 | 2 | 0 |
| 4 | 2 | 72 | 33 | 1 | 1 | 0 |
| 4 | 1 | 58 | 21 | 1 | 1 | 0 |
| 4 | 2 | 27 | 28 | 1 | 1 | 0 |
| 4 | 1 | 43 | 31 | 1 | 1 | 0 |
| 4 | 2 | 20 | 32 | 1 | 1 | 0 |
| 4 | 2 | 62 | 22 | 1 | 1 | 0 |
| 4 | 2 | 74 | 22 | 1 | 1 | 0 |
| 4 | 1 | 72 | 22 | 1 | 1 | 0 |
| 4 | 2 | 91 | 19 | 1 | 1 | 0 |
| 4 | 2 | 73 | 24 | 1 | 1 | 0 |
| 4 | 2 | 71 | 28 | 1 | 2 | 0 |
| 4 | 2 | 30 | 31 | 1 | 1 | 0 |
| 4 | 2 | 32 | 20 | 1 | 1 | 0 |
| 4 | 2 | 63 | 34 | 1 | 2 | 0 |
| 4 | 2 | 64 | 19 | 1 | 2 | 0 |
| 4 | 1 | 35 | 20 | 1 | 1 | 0 |
| 4 | 2 | 45 | 27 | 1 | 1 | 0 |
| 4 | 1 | 39 | 33 | 1 | 1 | 0 |
| 4 | 2 | 26 | 21 | 1 | 1 | 0 |
| 4 | 2 | 46 | 22 | 1 | 1 | 0 |
| 4 | 2 | 36 | 24 | 1 | 1 | 0 |
| 4 | 2 | 33 | 30 | 1 | 1 | 0 |
| 4 | 1 | 84 | 32 | 1 | 1 | 0 |
| 4 | 2 | 54 | 33 | 1 | 1 | 0 |
| 4 | 1 | 32 | 33 | 1 | 1 | 0 |
| 4 | 1 | 65 | 28 | 1 | 2 | 0 |
| 4 | 1 | 32 | 21 | 1 | 1 | 0 |
| 4 | 1 | 44 | 23 | 1 | 1 | 0 |
| 4 | 1 | 26 | 28 | 1 | 1 | 0 |
| 4 | 2 | 52 | 17 | 1 | 1 | 0 |
| 4 | 2 | 42 | 20 | 1 | 1 | 0 |
| 4 | 2 | 36 | 22 | 1 | 1 | 0 |
| 4 | 2 | 42 | 29 | 1 | 1 | 0 |
| 4 | 1 | 59 | 29 | 1 | 1 | 0 |
| 4 | 2 | 32 | 33 | 1 | 1 | 0 |
| 4 | 1 | 58 | 21 | 1 | 1 | 0 |
| 4 | 1 | 58 | 25 | 1 | 1 | 0 |
| 4 | 2 | 20 | 33 | 1 | 1 | 0 |
| 4 | 1 | 59 | 24 | 1 | 1 | 1 |
| 4 | 1 | 22 | 27 | 1 | 1 | 0 |
| 4 | 1 | 17 | 32 | 1 | 1 | 0 |
| 4 | 2 | 64 | 21 | 1 | 1 | 1 |
| 4 | 2 | 26 | 27 | 1 | 1 | 0 |

| | | | | | | |
|---|---|---|---|---|---|---|
| 4 | 1 | 63 | 31 | 1 | 1 | 0 |
| 4 | 1 | 70 | 19 | 1 | 1 | 1 |
| 4 | 2 | 44 | 24 | 1 | 1 | 1 |
| 4 | 2 | 70 | 25 | 1 | 1 | 1 |
| 4 | 2 | 68 | 24 | 1 | 1 | 0 |
| 4 | 1 | 78 | 18 | 1 | 2 | 0 |
| 4 | 1 | 45 | 20 | 1 | 2 | 0 |
| 4 | 1 | 51 | 26 | 1 | 2 | 0 |
| 4 | 1 | 86 | 27 | 1 | 2 | 0 |
| 4 | 2 | 30 | 25 | 1 | 1 | 0 |
| 4 | 1 | 59 | 27 | 1 | 1 | 0 |
| 4 | 1 | 22 | 33 | 1 | 1 | 1 |
| 4 | 1 | 41 | 23 | 1 | 1 | 0 |
| 4 | 1 | 37 | 28 | 1 | 1 | 0 |
| 4 | 1 | 43 | 29 | 1 | 1 | 0 |
| 4 | 1 | 60 | 22 | 1 | 1 | 0 |
| 4 | 1 | 39 | 26 | 1 | 1 | 0 |
| 4 | 1 | 87 | 20 | 1 | 1 | 1 |
| 4 | 1 | 15 | 27 | 1 | 1 | 0 |
| 4 | 2 | 87 | 33 | 1 | 2 | 0 |
| 4 | 1 | 37 | 23 | 1 | 1 | 0 |
| 4 | 2 | 20 | 23 | 1 | 1 | 0 |
| 4 | 1 | 68 | 23 | 1 | 1 | 0 |
| 4 | 2 | 80 | 23 | 1 | 1 | 0 |
| 4 | 1 | 50 | 25 | 1 | 1 | 0 |
| 4 | 2 | 77 | 32 | 1 | 1 | 0 |
| 4 | 1 | 91 | 32 | 1 | 1 | 0 |
| 4 | 2 | 61 | 34 | 1 | 1 | 0 |
| 4 | 1 | 73 | 35 | 1 | 1 | 1 |
| 4 | 1 | 27 | 22 | 1 | 1 | 0 |
| 4 | 2 | 45 | 23 | 1 | 1 | 0 |
| 4 | 2 | 72 | 27 | 1 | 1 | 0 |
| 4 | 1 | 65 | 28 | 1 | 1 | 0 |
| 4 | 2 | 35 | 21 | 1 | 1 | 0 |
| 4 | 2 | 16 | 27 | 1 | 1 | 0 |
| 4 | 1 | 43 | 22 | 1 | 1 | 0 |
| 4 | 2 | 82 | 27 | 1 | 1 | 0 |
| 4 | 2 | 65 | 25 | 1 | 1 | 0 |
| 4 | 2 | 28 | 30 | 1 | 1 | 0 |
| 4 | 1 | 79 | 24 | 1 | 2 | 0 |
| 4 | 2 | 45 | 31 | 1 | 1 | 0 |
| 4 | 2 | 50 | 26 | 1 | 1 | 0 |
| 4 | 2 | 47 | 32 | 1 | 1 | 0 |
| 4 | 1 | 57 | 22 | 1 | 1 | 0 |
| 4 | 2 | 18 | 25 | 1 | 1 | 0 |
| 4 | 2 | 50 | 32 | 1 | 1 | 0 |
| 4 | 1 | 43 | 32 | 1 | 1 | 0 |
| 4 | 2 | 30 | 30 | 1 | 1 | 0 |
| 4 | 2 | 50 | 27 | 1 | 1 | 0 |
| 4 | 1 | 11 | 24 | 1 | 1 | 0 |

| | | | | | | |
|---|---|---|---|---|---|---|
| 4 | 1 | 66 | 27 | 1 | 1 | 0 |
| 4 | 1 | 53 | 29 | 1 | 1 | 0 |
| 4 | 2 | 69 | 30 | 1 | 1 | 0 |
| 4 | 1 | 54 | 31 | 1 | 1 | 0 |
| 4 | 1 | 51 | 32 | 1 | 1 | 0 |
| 4 | 1 | 37 | 27 | 1 | 1 | 0 |
| 4 | 1 | 52 | 28 | 1 | 1 | 0 |
| 4 | 2 | 61 | 29 | 1 | 1 | 0 |
| 4 | 2 | 33 | 27 | 1 | 1 | 0 |
| 4 | 2 | 85 | 20 | 1 | 1 | 0 |
| 4 | 2 | 41 | 25 | 1 | 1 | 0 |
| 4 | 1 | 84 | 27 | 1 | 1 | 0 |
| 4 | 2 | 83 | 26 | 1 | 1 | 0 |
| 4 | 1 | 48 | 24 | 1 | 1 | 1 |
| 4 | 1 | 87 | 21 | 1 | 2 | 0 |
| 4 | 1 | 66 | 25 | 1 | 1 | 0 |
| 4 | 2 | 32 | 30 | 1 | 1 | 0 |
| 4 | 2 | 59 | 33 | 1 | 1 | 0 |
| 4 | 1 | 55 | 28 | 1 | 2 | 0 |
| 4 | 1 | 43 | 20 | 1 | 1 | 1 |
| 4 | 1 | 20 | 15 | 1 | 1 | 0 |
| 4 | 1 | 32 | 26 | 1 | 1 | 0 |
| 4 | 2 | 50 | 19 | 1 | 1 | 0 |
| 4 | 2 | 24 | 21 | 1 | 1 | 0 |
| 4 | 2 | 49 | 23 | 1 | 1 | 0 |
| 4 | 1 | 51 | 24 | 1 | 1 | 0 |
| 4 | 2 | 54 | 27 | 1 | 1 | 0 |
| 4 | 1 | 35 | 36 | 1 | 1 | 0 |
| 4 | 2 | 57 | 25 | 1 | 1 | 0 |
| 4 | 1 | 59 | 22 | 1 | 1 | 0 |
| 4 | 1 | 50 | 23 | 1 | 1 | 0 |
| 4 | 1 | 45 | 32 | 1 | 1 | 0 |
| 4 | 1 | 23 | 34 | 1 | 1 | 0 |
| 4 | 1 | 46 | 22 | 1 | 1 | 0 |
| 4 | 2 | 19 | 34 | 1 | 1 | 0 |
| 4 | 1 | 62 | 33 | 1 | 2 | 0 |
| 4 | 2 | 25 | 27 | 1 | 1 | 0 |
| 4 | 1 | 61 | 24 | 1 | 1 | 0 |
| 4 | 2 | 55 | 26 | 1 | 1 | 0 |
| 4 | 1 | 60 | 22 | 1 | 1 | 0 |
| 4 | 1 | 36 | 24 | 1 | 1 | 1 |
| 4 | 2 | 40 | 27 | 1 | 1 | 1 |
| 4 | 1 | 15 | 27 | 1 | 1 | 1 |
| 4 | 1 | 48 | 33 | 1 | 1 | 0 |
| 4 | 2 | 27 | 20 | 1 | 1 | 0 |
| 4 | 1 | 55 | 23 | 1 | 1 | 0 |
| 4 | 1 | 57 | 24 | 1 | 1 | 0 |
| 4 | 1 | 80 | 27 | 1 | 1 | 0 |
| 4 | 2 | 24 | 27 | 1 | 1 | 0 |
| 4 | 1 | 33 | 27 | 1 | 1 | 0 |

| | | | | | | |
|---|---|---|---|---|---|---|
| 4 | 1 | 56 | 31 | 1 | 1 | 0 |
| 4 | 2 | 52 | 33 | 1 | 1 | 0 |
| 4 | 2 | 25 | 29 | 1 | 1 | 0 |
| 4 | 2 | 28 | 30 | 1 | 1 | 0 |
| 4 | 1 | 44 | 31 | 1 | 1 | 0 |
| 4 | 2 | 9 | 32 | 1 | 1 | 0 |
| 4 | 1 | 65 | 25 | 1 | 1 | 0 |
| 4 | 2 | 64 | 26 | 1 | 1 | 0 |
| 4 | 1 | 36 | 23 | 1 | 1 | 0 |
| 4 | 2 | 14 | 29 | 1 | 1 | 0 |
| 4 | 1 | 37 | 34 | 1 | 1 | 0 |
| 4 | 1 | 55 | 35 | 1 | 1 | 0 |
| 4 | 2 | 15 | 27 | 1 | 1 | 0 |
| 4 | 1 | 64 | 32 | 1 | 1 | 0 |
| 4 | 2 | 90 | 33 | 1 | 1 | 0 |
| 4 | 1 | 83 | 20 | 1 | 2 | 0 |
| 4 | 2 | 38 | 23 | 1 | 1 | 0 |
| 4 | 1 | 37 | 30 | 1 | 1 | 0 |
| 4 | 2 | 22 | 22 | 1 | 1 | 0 |
| 4 | 1 | 41 | 25 | 1 | 1 | 0 |
| 4 | 2 | 79 | 24 | 1 | 1 | 0 |
| 4 | 2 | 47 | 19 | 1 | 1 | 0 |
| 4 | 1 | 81 | 22 | 1 | 1 | 0 |
| 4 | 1 | 91 | 22 | 1 | 1 | 0 |
| 4 | 1 | 95 | 22 | 1 | 1 | 0 |
| 4 | 1 | 79 | 23 | 1 | 1 | 0 |
| 4 | 1 | 46 | 31 | 1 | 1 | 0 |
| 4 | 2 | 59 | 31 | 1 | 1 | 0 |
| 4 | 2 | 64 | 21 | 1 | 1 | 0 |
| 4 | 1 | 86 | 35 | 1 | 1 | 0 |
| 4 | 2 | 32 | 20 | 1 | 1 | 0 |
| 4 | 1 | 37 | 30 | 1 | 1 | 0 |
| 4 | 2 | 64 | 26 | 1 | 1 | 0 |
| 4 | 1 | 19 | 29 | 1 | 1 | 0 |
| 4 | 2 | 65 | 30 | 1 | 1 | 0 |
| 4 | 2 | 34 | 22 | 1 | 1 | 0 |
| 4 | 2 | 23 | 31 | 1 | 1 | 0 |
| 4 | 1 | 24 | 35 | 1 | 1 | 0 |
| 4 | 1 | 62 | 21 | 1 | 1 | 0 |
| 4 | 2 | 65 | 24 | 1 | 1 | 0 |
| 4 | 1 | 90 | 24 | 1 | 1 | 0 |
| 4 | 2 | 61 | 28 | 1 | 1 | 0 |
| 4 | 1 | 59 | 30 | 1 | 1 | 0 |
| 4 | 1 | 47 | 31 | 1 | 1 | 0 |
| 4 | 1 | 25 | 31 | 1 | 1 | 0 |
| 4 | 1 | 38 | 25 | 1 | 1 | 1 |
| 4 | 1 | 83 | 31 | 1 | 2 | 0 |
| 4 | 2 | 50 | 26 | 1 | 1 | 0 |
| 4 | 1 | 20 | 28 | 1 | 1 | 0 |
| 4 | 2 | 38 | 32 | 1 | 1 | 0 |

| | | | | | | |
|---|---|---|---|---|---|---|
| 4 | 2 | 47 | 27 | 1 | 1 | 0 |
| 4 | 1 | 46 | 27 | 1 | 1 | 0 |
| 4 | 2 | 41 | 34 | 1 | 1 | 0 |
| 4 | 2 | 72 | 21 | 1 | 1 | 0 |
| 4 | 1 | 81 | 26 | 1 | 1 | 0 |
| 4 | 2 | 32 | 34 | 1 | 1 | 0 |
| 4 | 1 | 51 | 35 | 1 | 1 | 0 |
| 4 | 2 | 25 | 20 | 1 | 1 | 0 |
| 4 | 2 | 78 | 25 | 1 | 1 | 0 |
| 4 | 2 | 34 | 29 | 1 | 1 | 0 |
| 4 | 1 | 49 | 18 | 1 | 1 | 0 |
| 4 | 1 | 35 | 28 | 1 | 1 | 0 |
| 4 | 1 | 38 | 35 | 1 | 1 | 0 |
| 4 | 1 | 17 | 26 | 1 | 1 | 0 |
| 4 | 1 | 28 | 22 | 1 | 1 | 0 |
| 4 | 2 | 71 | 27 | 1 | 2 | 0 |
| 4 | 2 | 15 | 29 | 1 | 1 | 0 |
| 4 | 1 | 58 | 30 | 1 | 1 | 0 |
| 4 | 1 | 19 | 30 | 1 | 1 | 0 |
| 4 | 2 | 51 | 34 | 1 | 2 | 0 |
| 4 | 1 | 49 | 21 | 1 | 1 | 0 |
| 4 | 2 | 47 | 23 | 1 | 1 | 1 |
| 4 | 1 | 32 | 35 | 1 | 1 | 0 |
| 4 | 1 | 60 | 20 | 1 | 1 | 1 |
| 4 | 1 | 35 | 23 | 1 | 1 | 1 |
| 4 | 2 | 40 | 23 | 1 | 1 | 1 |
| 4 | 1 | 27 | 25 | 1 | 1 | 1 |
| 4 | 1 | 30 | 27 | 1 | 1 | 1 |
| 4 | 1 | 80 | 17 | 1 | 1 | 0 |
| 4 | 2 | 53 | 24 | 1 | 1 | 0 |
| 4 | 1 | 37 | 23 | 1 | 1 | 1 |
| 4 | 2 | 41 | 28 | 1 | 1 | 1 |
| 4 | 1 | 22 | 33 | 1 | 1 | 0 |
| 4 | 2 | 2 | 25 | 1 | 1 | 0 |
| 4 | 1 | 54 | 33 | 1 | 1 | 0 |
| 4 | 1 | 55 | 33 | 1 | 1 | 0 |
| 4 | 1 | 47 | 32 | 1 | 1 | 1 |
| 4 | 2 | 47 | 34 | 1 | 1 | 1 |
| 4 | 1 | 27 | 34 | 1 | 1 | 0 |
| 4 | 1 | 63 | 28 | 1 | 1 | 1 |
| 4 | 1 | 53 | 28 | 1 | 1 | 1 |
| 4 | 2 | 57 | 34 | 1 | 1 | 1 |
| 4 | 2 | 35 | 26 | 1 | 1 | 0 |
| 4 | 1 | 58 | 27 | 1 | 1 | 0 |
| 4 | 1 | 51 | 33 | 1 | 1 | 0 |
| 4 | 1 | 27 | 34 | 1 | 1 | 0 |
| 4 | 2 | 20 | 35 | 1 | 1 | 0 |
| 4 | 2 | 59 | 36 | 1 | 1 | 0 |
| 4 | 1 | 73 | 34 | 1 | 1 | 1 |
| 4 | 1 | 30 | 25 | 1 | 1 | 1 |

| | | | | | | |
|---|---|---|---|---|---|---|
| 4 | 2 | 64 | 33 | 1 | 1 | 0 |
| 4 | 1 | 54 | 25 | 1 | 1 | 0 |
| 4 | 2 | 52 | 25 | 1 | 1 | 0 |
| 4 | 1 | 63 | 34 | 1 | 1 | 0 |
| 4 | 1 | 22 | 28 | 1 | 1 | 1 |
| 4 | 1 | 6 | 25 | 1 | 1 | 0 |
| 4 | 1 | 40 | 27 | 1 | 1 | 0 |
| 4 | 2 | 16 | 30 | 1 | 1 | 0 |
| 4 | 2 | 58 | 30 | 1 | 1 | 0 |
| 4 | 2 | 90 | 28 | 1 | 1 | 0 |
| 4 | 2 | 17 | 29 | 1 | 1 | 0 |
| 4 | 1 | 48 | 22 | 1 | 1 | 0 |
| 4 | 2 | 49 | 27 | 1 | 1 | 0 |
| 4 | 1 | 35 | 34 | 1 | 1 | 1 |
| 4 | 2 | 19 | 21 | 1 | 1 | 0 |
| 4 | 2 | 17 | 26 | 1 | 1 | 0 |
| 4 | 2 | 44 | 28 | 1 | 1 | 0 |
| 4 | 1 | 39 | 29 | 1 | 1 | 0 |
| 4 | 1 | 58 | 34 | 1 | 1 | 0 |
| 4 | 1 | 58 | 34 | 1 | 1 | 0 |
| 4 | 1 | 80 | 21 | 1 | 1 | 0 |
| 4 | 2 | 44 | 22 | 1 | 1 | 0 |
| 4 | 2 | 19 | 22 | 1 | 1 | 0 |
| 5 | 1 | 17 | 24 | 1 | 1 | 0 |
| 5 | 1 | 19 | 19 | 1 | 1 | 0 |
| 5 | 2 | 44 | 30 | 1 | 1 | 0 |
| 5 | 2 | 56 | 26 | 1 | 1 | 0 |
| 5 | 1 | 28 | 17 | 1 | 1 | 0 |
| 5 | 1 | 3 | 27 | 1 | 1 | 1 |
| 5 | 2 | 23 | 24 | 1 | 1 | 0 |
| 5 | 2 | 40 | 29 | 1 | 2 | 1 |
| 6 | 1 | 69 | 28 | 1 | 2 | 0 |
| 6 | 2 | 62 | 28 | 1 | 1 | 0 |
| 6 | 1 | 73 | 24 | 1 | 1 | 0 |
| 6 | 1 | 28 | 23 | 1 | 1 | 0 |
| 6 | 1 | 24 | 19 | 1 | 1 | 0 |
| 6 | 1 | 25 | 23 | 1 | 1 | 0 |
| 6 | 1 | 55 | 23 | 1 | 1 | 0 |
| 6 | 1 | 62 | 29 | 1 | 1 | 0 |
| 6 | 1 | 67 | 21 | 1 | 1 | 1 |
| 6 | 2 | 34 | 27 | 1 | 1 | 1 |
| 6 | 1 | 52 | 26 | 1 | 1 | 0 |
| 6 | 1 | 36 | 27 | 1 | 1 | 0 |
| 6 | 2 | 67 | 32 | 1 | 1 | 0 |
| 6 | 2 | 29 | 21 | 1 | 1 | 0 |
| 6 | 1 | 25 | 23 | 1 | 1 | 0 |
| 6 | 1 | 68 | 26 | 1 | 1 | 0 |
| 6 | 2 | 7 | 20 | 1 | 1 | 0 |
| 6 | 2 | 3 | 21 | 1 | 1 | 0 |
| 6 | 2 | 19 | 24 | 1 | 1 | 0 |

| | | | | | | |
|---|---|---|---|---|---|---|
| 6 | 2 | 58 | 25 | 1 | 1 | 0 |
| 6 | 2 | 7 | 29 | 1 | 1 | 0 |
| 6 | 1 | 44 | 30 | 1 | 1 | 0 |
| 6 | 2 | 22 | 30 | 1 | 1 | 1 |
| 6 | 2 | 25 | 26 | 1 | 1 | 0 |
| 6 | 2 | 23 | 32 | 1 | 1 | 0 |
| 6 | 2 | 70 | 26 | 1 | 2 | 0 |
| 6 | 2 | 33 | 27 | 1 | 1 | 0 |
| 6 | 1 | 34 | 24 | 1 | 1 | 1 |
| 6 | 1 | 56 | 21 | 1 | 1 | 1 |
| 6 | 2 | 26 | 30 | 1 | 1 | 0 |
| 6 | 1 | 79 | 22 | 1 | 2 | 0 |
| 6 | 2 | 31 | 25 | 1 | 1 | 0 |
| 6 | 1 | 24 | 27 | 1 | 1 | 0 |
| 6 | 1 | 61 | 32 | 1 | 1 | 0 |
| 6 | 1 | 28 | 18 | 1 | 1 | 1 |
| 6 | 1 | 60 | 24 | 1 | 1 | 0 |
| 6 | 1 | 52 | 24 | 1 | 1 | 0 |
| 6 | 1 | 52 | 26 | 1 | 2 | 0 |
| 6 | 2 | 60 | 20 | 1 | 1 | 0 |
| 6 | 1 | 41 | 24 | 1 | 1 | 0 |
| 6 | 1 | 80 | 28 | 1 | 2 | 0 |
| 6 | 2 | 81 | 24 | 1 | 1 | 0 |
| 6 | 2 | 43 | 25 | 1 | 1 | 0 |
| 6 | 2 | 60 | 26 | 1 | 2 | 0 |
| 6 | 1 | 47 | 23 | 1 | 1 | 0 |
| 6 | 2 | 58 | 28 | 1 | 1 | 0 |
| 6 | 1 | 68 | 26 | 1 | 2 | 0 |
| 6 | 1 | 28 | 18 | 1 | 1 | 0 |
| 6 | 1 | 35 | 23 | 1 | 1 | 1 |
| 6 | 2 | 49 | 25 | 1 | 1 | 0 |
| 6 | 2 | 29 | 29 | 1 | 1 | 1 |
| 6 | 1 | 63 | 30 | 1 | 1 | 0 |
| 6 | 2 | 40 | 30 | 1 | 2 | 1 |
| 6 | 1 | 62 | 27 | 1 | 1 | 0 |
| 6 | 1 | 30 | 24 | 1 | 1 | 1 |
| 6 | 1 | 59 | 24 | 1 | 1 | 0 |
| 6 | 2 | 58 | 24 | 1 | 2 | 0 |
| 6 | 2 | 35 | 27 | 1 | 1 | 0 |
| 6 | 1 | 61 | 24 | 1 | 1 | 1 |
| 6 | 2 | 63 | 27 | 1 | 1 | 1 |
| 6 | 2 | 48 | 26 | 1 | 1 | 0 |
| 6 | 1 | 83 | 28 | 1 | 2 | 1 |
| 6 | 2 | 35 | 20 | 1 | 1 | 0 |
| 6 | 1 | 23 | 30 | 1 | 1 | 0 |
| 6 | 2 | 10 | 25 | 1 | 1 | 1 |
| 6 | 1 | 31 | 28 | 1 | 1 | 1 |
| 6 | 1 | 27 | 29 | 1 | 1 | 1 |
| 6 | 2 | 74 | 18 | 1 | 1 | 0 |
| 6 | 2 | 46 | 32 | 1 | 1 | 0 |

| | | | | | | |
|---|---|---|---|---|---|---|
| 6 | 2 | 36 | 29 | 1 | 1 | 0 |
| 6 | 2 | 44 | 25 | 1 | 1 | 1 |
| 6 | 1 | 26 | 30 | 1 | 1 | 1 |
| 6 | 1 | 70 | 17 | 1 | 1 | 0 |
| 6 | 1 | 40 | 26 | 1 | 1 | 0 |
| 6 | 2 | 73 | 30 | 1 | 1 | 0 |
| 6 | 1 | 60 | 22 | 1 | 1 | 1 |
| 6 | 2 | 68 | 23 | 1 | 1 | 1 |
| 6 | 1 | 23 | 28 | 1 | 1 | 1 |
| 6 | 1 | 36 | 28 | 1 | 1 | 0 |
| 6 | 1 | 58 | 24 | 1 | 1 | 1 |
| 6 | 1 | 16 | 27 | 1 | 1 | 1 |
| 6 | 2 | 40 | 32 | 1 | 1 | 1 |
| 6 | 2 | 4 | 24 | 1 | 1 | 0 |
| 6 | 2 | 59 | 28 | 1 | 1 | 0 |
| 6 | 1 | 53 | 26 | 1 | 1 | 0 |
| 6 | 1 | 39 | 18 | 1 | 1 | 0 |
| 6 | 2 | 21 | 25 | 1 | 1 | 0 |
| 6 | 1 | 26 | 27 | 1 | 1 | 0 |
| 6 | 1 | 49 | 31 | 1 | 1 | 0 |
| 6 | 2 | 50 | 18 | 1 | 1 | 1 |
| 6 | 1 | 49 | 22 | 1 | 1 | 0 |
| 6 | 1 | 21 | 19 | 1 | 1 | 1 |
| 6 | 2 | 51 | 31 | 1 | 1 | 0 |
| 6 | 2 | 19 | 25 | 1 | 1 | 0 |
| 6 | 1 | 83 | 32 | 1 | 1 | 1 |
| 6 | 1 | 26 | 24 | 1 | 1 | 0 |
| 6 | 2 | 48 | 24 | 1 | 1 | 0 |
| 6 | 2 | 61 | 24 | 1 | 1 | 0 |
| 6 | 2 | 87 | 28 | 1 | 1 | 0 |
| 6 | 2 | 8 | 29 | 1 | 1 | 0 |
| 6 | 2 | 83 | 30 | 1 | 1 | 0 |
| 6 | 2 | 67 | 30 | 1 | 1 | 0 |
| 6 | 1 | 11 | 32 | 1 | 1 | 0 |
| 6 | 1 | 46 | 32 | 1 | 1 | 0 |
| 6 | 1 | 49 | 27 | 1 | 1 | 0 |
| 6 | 1 | 24 | 23 | 1 | 1 | 0 |
| 6 | 2 | 29 | 26 | 1 | 1 | 0 |
| 6 | 1 | 27 | 32 | 1 | 1 | 0 |
| 6 | 2 | 76 | 32 | 1 | 1 | 0 |
| 6 | 2 | 21 | 22 | 1 | 1 | 0 |
| 6 | 1 | 87 | 32 | 1 | 1 | 0 |
| 6 | 2 | 26 | 24 | 1 | 1 | 0 |
| 6 | 2 | 55 | 23 | 1 | 1 | 0 |
| 6 | 1 | 51 | 23 | 1 | 1 | 0 |
| 6 | 2 | 54 | 24 | 1 | 1 | 0 |
| 6 | 2 | 8 | 22 | 1 | 1 | 1 |
| 6 | 1 | 62 | 18 | 1 | 1 | 0 |
| 6 | 1 | 24 | 24 | 1 | 1 | 1 |
| 6 | 2 | 8 | 24 | 1 | 1 | 0 |

| | | | | | | |
|---|---|---|---|---|---|---|
| 6 | 1 | 49 | 17 | 1 | 1 | 0 |
| 6 | 2 | 66 | 28 | 1 | 1 | 1 |
| 6 | 1 | 50 | 29 | 1 | 1 | 0 |
| 6 | 1 | 64 | 27 | 1 | 1 | 0 |
| 6 | 2 | 38 | 24 | 1 | 1 | 1 |
| 6 | 1 | 64 | 27 | 1 | 1 | 1 |
| 6 | 1 | 59 | 28 | 1 | 1 | 0 |
| 6 | 2 | 79 | 26 | 1 | 2 | 0 |
| 6 | 1 | 44 | 24 | 1 | 1 | 1 |
| 6 | 2 | 23 | 20 | 1 | 1 | 0 |
| 6 | 1 | 29 | 32 | 1 | 1 | 0 |
| 6 | 1 | 31 | 26 | 1 | 1 | 1 |
| 6 | 1 | 85 | 31 | 1 | 2 | 0 |
| 6 | 2 | 86 | 27 | 1 | 1 | 0 |
| 6 | 2 | 30 | 27 | 1 | 1 | 1 |
| 6 | 2 | 66 | 27 | 1 | 1 | 0 |
| 6 | 2 | 38 | 23 | 1 | 1 | 0 |
| 6 | 1 | 62 | 24 | 1 | 1 | 0 |
| 6 | 1 | 67 | 25 | 1 | 1 | 1 |
| 6 | 2 | 61 | 25 | 1 | 1 | 0 |
| 6 | 2 | 52 | 19 | 1 | 1 | 1 |
| 6 | 1 | 65 | 30 | 1 | 1 | 1 |
| 6 | 2 | 82 | 21 | 1 | 1 | 0 |
| 6 | 1 | 41 | 19 | 1 | 1 | 0 |
| 6 | 2 | 48 | 25 | 1 | 1 | 0 |
| 6 | 2 | 34 | 22 | 1 | 1 | 1 |
| 6 | 1 | 48 | 22 | 1 | 2 | 1 |
| 6 | 2 | 37 | 21 | 1 | 1 | 1 |
| 6 | 1 | 24 | 32 | 1 | 1 | 1 |
| 6 | 1 | 46 | 30 | 1 | 1 | 0 |
| 6 | 2 | 44 | 31 | 1 | 1 | 0 |
| 6 | 2 | 48 | 22 | 1 | 1 | 0 |
| 6 | 1 | 62 | 29 | 1 | 1 | 0 |
| 6 | 2 | 50 | 29 | 1 | 1 | 0 |
| 6 | 1 | 71 | 19 | 1 | 1 | 0 |
| 6 | 1 | 94 | 19 | 1 | 1 | 0 |
| 6 | 1 | 82 | 21 | 1 | 1 | 0 |
| 6 | 1 | 83 | 26 | 1 | 1 | 0 |
| 6 | 1 | 39 | 28 | 1 | 1 | 0 |
| 6 | 2 | 48 | 28 | 1 | 1 | 1 |
| 6 | 1 | 81 | 22 | 1 | 1 | 0 |
| 6 | 2 | 78 | 28 | 1 | 2 | 0 |
| 6 | 1 | 37 | 32 | 1 | 1 | 0 |
| 6 | 1 | 38 | 20 | 1 | 1 | 1 |
| 6 | 2 | 86 | 21 | 1 | 1 | 0 |
| 6 | 2 | 23 | 30 | 1 | 1 | 0 |
| 6 | 2 | 53 | 32 | 1 | 1 | 0 |
| 6 | 1 | 58 | 27 | 1 | 1 | 0 |
| 6 | 1 | 28 | 20 | 1 | 1 | 0 |
| 6 | 2 | 61 | 24 | 1 | 1 | 1 |

| | | | | | | |
|---|---|---|---|---|---|---|
| 6 | 1 | 68 | 28 | 1 | 2 | 0 |
| 6 | 1 | 41 | 28 | 1 | 2 | 1 |
| 6 | 1 | 41 | 24 | 1 | 1 | 1 |
| 6 | 1 | 37 | 31 | 1 | 1 | 0 |
| 6 | 1 | 47 | 32 | 1 | 1 | 0 |
| 6 | 1 | 37 | 24 | 1 | 1 | 0 |
| 6 | 2 | 62 | 22 | 1 | 1 | 0 |
| 6 | 2 | 58 | 26 | 1 | 1 | 0 |
| 6 | 2 | 45 | 27 | 1 | 1 | 0 |
| 6 | 1 | 24 | 24 | 1 | 1 | 1 |
| 6 | 1 | 21 | 32 | 1 | 1 | 1 |
| 6 | 1 | 51 | 28 | 1 | 1 | 0 |
| 6 | 1 | 38 | 24 | 1 | 1 | 1 |
| 6 | 1 | 36 | 25 | 1 | 1 | 0 |
| 6 | 2 | 24 | 31 | 1 | 1 | 0 |
| 6 | 2 | 58 | 32 | 1 | 1 | 0 |
| 6 | 1 | 29 | 24 | 1 | 1 | 1 |
| 6 | 1 | 66 | 23 | 1 | 1 | 0 |
| 6 | 1 | 44 | 30 | 1 | 1 | 0 |
| 6 | 1 | 57 | 28 | 1 | 1 | 0 |
| 6 | 2 | 67 | 30 | 1 | 1 | 0 |
| 6 | 2 | 70 | 21 | 1 | 1 | 1 |
| 6 | 1 | 68 | 24 | 1 | 1 | 1 |
| 6 | 1 | 66 | 24 | 1 | 1 | 1 |
| 6 | 2 | 6 | 28 | 1 | 1 | 1 |
| 6 | 1 | 4 | 30 | 1 | 1 | 1 |
| 6 | 2 | 22 | 31 | 1 | 1 | 0 |
| 6 | 1 | 25 | 31 | 1 | 1 | 0 |
| 6 | 2 | 50 | 17 | 1 | 1 | 0 |
| 6 | 1 | 46 | 22 | 1 | 1 | 0 |
| 6 | 1 | 19 | 20 | 1 | 1 | 0 |
| 6 | 2 | 63 | 25 | 1 | 1 | 0 |
| 6 | 1 | 37 | 26 | 1 | 1 | 0 |
| 6 | 1 | 63 | 28 | 1 | 1 | 0 |
| 6 | 1 | 86 | 29 | 1 | 2 | 0 |
| 6 | 1 | 27 | 30 | 1 | 1 | 1 |
| 6 | 2 | 34 | 21 | 1 | 1 | 0 |
| 6 | 2 | 37 | 26 | 1 | 1 | 0 |
| 6 | 1 | 62 | 19 | 1 | 1 | 1 |
| 6 | 2 | 62 | 19 | 1 | 1 | 1 |
| 6 | 1 | 37 | 21 | 1 | 1 | 1 |
| 6 | 1 | 25 | 29 | 1 | 1 | 0 |
| 6 | 1 | 59 | 29 | 1 | 1 | 0 |
| 6 | 1 | 34 | 32 | 1 | 1 | 0 |
| 6 | 1 | 33 | 32 | 1 | 1 | 1 |
| 6 | 1 | 68 | 32 | 1 | 1 | 0 |
| 6 | 1 | 91 | 21 | 1 | 1 | 0 |
| 6 | 1 | 42 | 22 | 1 | 1 | 0 |
| 6 | 2 | 32 | 24 | 1 | 1 | 0 |
| 6 | 1 | 16 | 27 | 1 | 1 | 0 |

| | | | | | | |
|---|---|---|---|---|---|---|
| 6 | 2 | 31 | 29 | 1 | 1 | 0 |
| 6 | 2 | 11 | 31 | 1 | 1 | 0 |
| 6 | 2 | 13 | 32 | 1 | 1 | 0 |
| 6 | 2 | 32 | 23 | 1 | 1 | 0 |
| 6 | 2 | 68 | 21 | 1 | 1 | 0 |
| 6 | 1 | 68 | 32 | 1 | 1 | 0 |
| 6 | 1 | 71 | 25 | 1 | 2 | 0 |
| 6 | 1 | 31 | 20 | 1 | 1 | 1 |
| 6 | 2 | 58 | 30 | 1 | 2 | 0 |
| 6 | 2 | 75 | 31 | 1 | 1 | 0 |
| 6 | 2 | 32 | 26 | 1 | 1 | 1 |
| 6 | 1 | 52 | 28 | 1 | 1 | 0 |
| 6 | 2 | 49 | 29 | 1 | 1 | 0 |
| 6 | 2 | 56 | 30 | 1 | 1 | 0 |
| 6 | 2 | 24 | 22 | 1 | 1 | 1 |
| 6 | 1 | 65 | 31 | 1 | 1 | 0 |
| 6 | 1 | 45 | 22 | 1 | 1 | 0 |
| 6 | 2 | 49 | 31 | 1 | 1 | 0 |
| 6 | 1 | 18 | 21 | 1 | 1 | 0 |
| 6 | 1 | 30 | 21 | 1 | 1 | 1 |
| 6 | 2 | 55 | 23 | 1 | 1 | 0 |
| 6 | 1 | 19 | 23 | 1 | 1 | 0 |
| 6 | 2 | 16 | 25 | 1 | 1 | 0 |
| 6 | 1 | 19 | 29 | 1 | 1 | 0 |
| 6 | 2 | 52 | 32 | 1 | 1 | 0 |
| 6 | 1 | 55 | 32 | 1 | 1 | 0 |
| 6 | 2 | 67 | 29 | 1 | 2 | 0 |
| 6 | 2 | 35 | 28 | 1 | 1 | 0 |
| 6 | 1 | 80 | 28 | 1 | 1 | 1 |
| 6 | 2 | 54 | 23 | 1 | 1 | 0 |
| 6 | 1 | 55 | 23 | 1 | 1 | 0 |
| 6 | 2 | 21 | 31 | 1 | 1 | 0 |
| 6 | 2 | 88 | 32 | 1 | 1 | 0 |
| 6 | 1 | 53 | 20 | 1 | 1 | 1 |
| 6 | 1 | 32 | 23 | 1 | 1 | 1 |
| 6 | 1 | 44 | 24 | 1 | 1 | 1 |
| 6 | 2 | 83 | 25 | 1 | 1 | 1 |
| 6 | 1 | 33 | 25 | 1 | 1 | 1 |
| 6 | 1 | 72 | 27 | 1 | 1 | 1 |
| 6 | 1 | 91 | 31 | 1 | 1 | 1 |
| 6 | 2 | 61 | 25 | 1 | 1 | 1 |
| 6 | 2 | 92 | 24 | 1 | 1 | 0 |
| 6 | 2 | 53 | 24 | 1 | 1 | 0 |
| 6 | 1 | 70 | 24 | 1 | 1 | 1 |
| 6 | 2 | 38 | 31 | 1 | 1 | 0 |
| 6 | 2 | 61 | 32 | 1 | 1 | 0 |
| 6 | 1 | 42 | 21 | 1 | 1 | 1 |
| 6 | 2 | 47 | 26 | 1 | 1 | 1 |
| 6 | 1 | 63 | 20 | 1 | 1 | 0 |
| 6 | 1 | 83 | 25 | 1 | 2 | 0 |

| | | | | | | |
|---|---|---|---|---|---|---|
| 6 | 2 | 82 | 26 | 1 | 2 | 0 |
| 6 | 2 | 8 | 25 | 1 | 1 | 1 |
| 6 | 1 | 43 | 32 | 1 | 1 | 0 |
| 6 | 1 | 40 | 32 | 1 | 1 | 0 |
| 6 | 2 | 66 | 21 | 1 | 1 | 1 |
| 6 | 1 | 67 | 24 | 1 | 1 | 1 |
| 6 | 1 | 43 | 26 | 1 | 1 | 0 |
| 6 | 2 | 57 | 27 | 1 | 1 | 0 |
| 6 | 2 | 43 | 32 | 1 | 1 | 0 |
| 6 | 2 | 26 | 29 | 1 | 1 | 0 |
| 6 | 2 | 31 | 28 | 1 | 1 | 1 |
| 6 | 2 | 59 | 32 | 1 | 1 | 1 |
| 6 | 1 | 41 | 20 | 1 | 1 | 0 |
| 6 | 1 | 62 | 24 | 1 | 1 | 0 |
| 6 | 1 | 47 | 28 | 1 | 1 | 0 |
| 6 | 1 | 31 | 25 | 1 | 1 | 0 |
| 6 | 2 | 33 | 20 | 1 | 1 | 1 |
| 6 | 1 | 55 | 23 | 1 | 1 | 0 |
| 6 | 2 | 56 | 31 | 1 | 1 | 0 |
| 6 | 1 | 29 | 25 | 1 | 1 | 0 |
| 6 | 2 | 73 | 24 | 1 | 1 | 1 |
| 6 | 1 | 64 | 25 | 1 | 1 | 1 |
| 6 | 1 | 58 | 24 | 1 | 1 | 1 |
| 6 | 1 | 82 | 23 | 1 | 1 | 0 |
| 6 | 1 | 18 | 22 | 1 | 1 | 0 |
| 6 | 1 | 48 | 25 | 1 | 1 | 0 |
| 6 | 2 | 65 | 27 | 1 | 1 | 1 |
| 6 | 2 | 73 | 32 | 1 | 1 | 0 |
| 6 | 2 | 9 | 24 | 1 | 1 | 0 |
| 6 | 2 | 11 | 28 | 1 | 1 | 0 |
| 6 | 1 | 30 | 23 | 1 | 1 | 0 |
| 6 | 2 | 69 | 25 | 1 | 1 | 0 |
| 6 | 2 | 43 | 29 | 1 | 1 | 0 |
| 6 | 1 | 82 | 21 | 1 | 2 | 0 |
| 6 | 2 | 92 | 18 | 1 | 2 | 0 |
| 6 | 2 | 25 | 31 | 1 | 1 | 0 |
| 7 | 2 | 63 | 27 | 1 | 1 | 0 |
| 7 | 1 | 25 | 27 | 1 | 1 | 0 |
| 7 | 2 | 30 | 26 | 1 | 1 | 0 |
| 7 | 2 | 60 | 32 | 1 | 1 | 1 |
| 7 | 1 | 8 | 28 | 1 | 1 | 0 |
| 7 | 2 | 10 | 30 | 1 | 1 | 0 |
| 7 | 1 | 53 | 22 | 1 | 1 | 1 |
| 7 | 2 | 53 | 23 | 1 | 1 | 1 |
| 7 | 1 | 48 | 30 | 1 | 1 | 1 |
| 7 | 2 | 49 | 25 | 1 | 1 | 1 |
| 7 | 1 | 49 | 28 | 1 | 1 | 1 |
| 7 | 1 | 14 | 18 | 1 | 1 | 1 |
| 7 | 1 | 65 | 24 | 1 | 1 | 0 |
| 7 | 1 | 25 | 25 | 1 | 1 | 0 |

| | | | | | | | |
|---|---|---|---|---|---|---|---|
| 7 | 2 | 38 | 25 | 1 | | 1 | 1 |
| 7 | 2 | 59 | 22 | 1 | | 1 | 0 |
| 7 | 2 | 27 | 27 | 1 | | 1 | 0 |
| 7 | 2 | 43 | 27 | 1 | | 1 | 1 |
| 7 | 2 | 24 | 29 | 1 | | 1 | 0 |
| 7 | 1 | 75 | 30 | 1 | | 1 | 0 |
| 7 | 1 | 16 | 22 | 1 | | 1 | 1 |
| 7 | 2 | 20 | 24 | 1 | | 1 | 1 |
| 7 | 1 | 49 | 26 | 1 | | 1 | 1 |
| 7 | 1 | 34 | 23 | 1 | | 1 | 1 |
| 8 | 1 | 58 | 18 | 1 | 2 | 1 | 1 |
| 8 | 1 | 32 | 30 | 1 | -2 | 1 | 1 |
| 8 | 2 | 60 | 24 | 1 | | 1 | 0 |
| 8 | 2 | 31 | 21 | 1 | | 1 | 0 |
| 8 | 1 | 63 | 23 | 1 | - | 2 | 0 |
| 8 | 2 | 42 | 32 | 1 | 0 | 1 | 0 |
| 8 | 2 | 20 | 32 | 1 | | 1 | 0 |
| 8 | 2 | 33 | 24 | 1 | 1 | 1 | 1 |
| 8 | 1 | 64 | 31 | 1 | | 1 | 1 |
| 8 | 1 | 59 | 31 | 1 | | 1 | 0 |
| 8 | 1 | 22 | 24 | 1 | 2 | 1 | 0 |
| 8 | 2 | 23 | 26 | 1 | 3 | 1 | 0 |
| 8 | 1 | 81 | 32 | 1 | | 1 | 0 |
| 8 | 1 | 38 | 24 | 1 | | 1 | 1 |
| 8 | 2 | 27 | 24 | 1 | | 1 | 0 |
| 8 | 2 | 6 | 24 | 1 | 2 | 1 | 1 |
| 8 | 1 | 69 | 18 | 1 | | 1 | 1 |
| 8 | 2 | 77 | 18 | 1 | | 1 | 1 |
| 8 | 2 | 29 | 22 | 1 | | 1 | 1 |
| 8 | 2 | 34 | 22 | 1 | | 1 | 1 |
| 8 | 2 | 41 | 24 | 1 | | 1 | 1 |
| 8 | 2 | 42 | 19 | 1 | | 1 | 0 |
| 8 | 2 | 58 | 24 | 1 | | 1 | 0 |
| 8 | 2 | 24 | 22 | 1 | 3 | 1 | 1 |
| 8 | 1 | 14 | 25 | 1 | | 1 | 1 |
| 8 | 2 | 51 | 25 | 1 | | 1 | 0 |
| 8 | 1 | 50 | 25 | 1 | | 1 | 0 |
| 8 | 1 | 38 | 29 | 1 | | 1 | 0 |
| 8 | 1 | 22 | 29 | 1 | | 1 | 1 |
| 8 | 1 | 31 | 32 | 1 | | 2 | 0 |
| 8 | 1 | 58 | 21 | 1 | 4 | 1 | 0 |
| 8 | 2 | 56 | 19 | 1 | | 1 | 0 |
| 8 | 1 | 25 | 27 | 1 | 4 | 1 | 0 |
| 8 | 2 | 64 | 28 | 1 | 4 | 1 | 0 |
| 8 | 1 | 41 | 23 | 1 | | 1 | 1 |
| 8 | 2 | 42 | 30 | 1 | | 1 | 1 |
| 8 | 2 | 75 | 26 | 1 | | 2 | 1 |
| 8 | 2 | 60 | 20 | 1 | | 1 | 0 |
| 8 | 1 | 22 | 23 | 1 | | 1 | 0 |
| 8 | 2 | 28 | 25 | 1 | | 1 | 0 |

| | | | | | | | |
|---|---|---|---|---|---|---|---|
| 8 | 2 | 44 | 20 | 1 | | 1 | 1 |
| 8 | 1 | 50 | 20 | 1 | | 1 | 1 |
| 8 | 2 | 8 | 32 | 1 | | 1 | 1 |
| 8 | 1 | 58 | 19 | 1 | 3 | 1 | 0 |
| 8 | 2 | 62 | 21 | 1 | 7 | 1 | 0 |
| 8 | 1 | 50 | 26 | 1 | 1 | 1 | 1 |
| 8 | 1 | 41 | 24 | 1 | | 1 | 0 |
| 8 | 2 | 21 | 21 | 1 | | 1 | 1 |
| 8 | 2 | 50 | 25 | 1 | | 1 | 0 |
| 8 | 2 | 27 | 22 | 1 | 4 | 1 | 1 |
| 8 | 1 | 83 | 23 | 1 | | 1 | 0 |
| 8 | 1 | 49 | 26 | 1 | | 1 | 0 |
| 8 | 2 | 19 | 31 | 1 | | 1 | 0 |
| 8 | 2 | 34 | 22 | 1 | 3 | 1 | 0 |
| 8 | 1 | 7 | 24 | 1 | 1 | 1 | 0 |
| 8 | 1 | 73 | 31 | 1 | 12 | 1 | 0 |
| 8 | 1 | 25 | 25 | 1 | | 1 | 0 |
| 8 | 2 | 55 | 21 | 1 | | 1 | 1 |
| 8 | 2 | 54 | 22 | 1 | 3 | 1 | 2 |
| 8 | 1 | 68 | 24 | 1 | | 1 | 1 |
| 8 | 2 | 36 | 27 | 1 | | 1 | 1 |
| 8 | 1 | 41 | 30 | 1 | | 1 | 1 |
| 8 | 1 | 67 | 23 | 1 | 3 | 1 | 0 |
| 8 | 2 | 67 | 27 | 1 | | 1 | 0 |
| 8 | 2 | 62 | 24 | 1 | | 1 | 1 |
| 8 | 1 | 28 | 19 | 1 | - | 1 | 1 |
| 8 | 1 | 72 | 22 | 1 | | 1 | 1 |
| 8 | 2 | 73 | 22 | 1 | | 1 | 1 |
| 8 | 2 | 2 | 32 | 1 | | 1 | 0 |
| 8 | 1 | 18 | 13 | 1 | | 1 | 1 |
| 8 | 2 | 50 | 23 | 1 | | 1 | 1 |
| 8 | 2 | 15 | 27 | 1 | | 1 | 1 |
| 8 | 2 | 48 | 25 | 1 | | 1 | 1 |
| 8 | 1 | 73 | 32 | 1 | | 2 | 0 |
| 8 | 2 | 55 | 31 | 1 | | 2 | 0 |
| 8 | 1 | 52 | 28 | 1 | | 1 | 1 |
| 8 | 2 | 66 | 19 | 1 | 3 | 1 | 0 |
| 8 | 2 | 35 | 28 | 1 | 1 | 1 | 1 |
| 8 | 2 | 39 | 31 | 1 | 2 | 1 | 1 |
| 8 | 1 | 30 | 20 | 1 | | 1 | 1 |
| 8 | 2 | 29 | 20 | 1 | | 1 | 1 |
| 8 | 2 | 49 | 22 | 1 | | 1 | 0 |
| 8 | 2 | 46 | 21 | 1 | | 1 | 0 |
| 8 | 1 | 61 | 22 | 1 | | 1 | 0 |
| 8 | 1 | 57 | 25 | 1 | | 1 | 0 |
| 8 | 2 | 34 | 21 | 1 | | 1 | 1 |
| 8 | 1 | 32 | 26 | 1 | | 1 | 1 |
| 8 | 1 | 22 | 26 | 1 | | 1 | 1 |
| 8 | 1 | 21 | 27 | 1 | | 1 | 1 |
| 8 | 1 | 60 | 21 | 1 | 4 | 1 | 1 |

| | | | | | | | |
|---|---|---|---|---|---|---|---|
| 8 | 1 | 23 | 22 | 1 | | 1 | 1 |
| 8 | 2 | 69 | 28 | 1 | | 1 | 1 |
| 8 | 2 | 63 | 27 | 1 | | 1 | 0 |
| 8 | 1 | 48 | 25 | 1 | 3 | 1 | 1 |
| 8 | 1 | 24 | 27 | 1 | | 1 | 1 |
| 8 | 2 | 20 | 24 | 1 | 2 | 1 | 0 |
| 8 | 2 | 58 | 24 | 1 | 2 | 1 | 0 |
| 8 | 2 | 23 | 30 | 1 | | 1 | 0 |
| 8 | 1 | 56 | 31 | 1 | | 1 | 0 |
| 8 | 2 | 63 | 21 | 1 | 0 | 1 | 1 |
| 8 | 2 | 7 | 32 | 1 | 2 | 1 | 1 |
| 8 | 1 | 81 | 24 | 1 | | 2 | 1 |
| 8 | 1 | 78 | 30 | 1 | | 2 | 1 |
| 8 | 2 | 5 | 29 | 1 | 1 | 1 | 0 |
| 8 | 2 | 53 | 24 | 1 | 3 | 1 | 0 |
| 8 | 1 | 15 | 29 | 1 | | 1 | 0 |
| 8 | 1 | 11 | 30 | 1 | | 1 | 0 |
| 8 | 1 | 54 | 19 | 1 | 4 | 1 | 1 |
| 8 | 1 | 43 | 23 | 1 | | 1 | 2 |
| 8 | 1 | 49 | 25 | 1 | 1 | 1 | 1 |
| 8 | 2 | 46 | 20 | 1 | 2 | 1 | 1 |
| 8 | 2 | 59 | 16 | 1 | | 1 | 1 |
| 8 | 2 | 19 | 32 | 1 | 1 | 1 | 0 |
| 8 | 2 | 51 | 31 | 1 | 1 | 1 | 0 |
| 8 | 1 | 36 | 25 | 1 | 7 | 1 | 0 |
| 8 | 1 | 26 | 17 | 1 | 0 | 1 | 1 |
| 8 | 2 | 42 | 21 | 1 | | 1 | 1 |
| 8 | 1 | 71 | 26 | 1 | | 1 | 1 |
| 8 | 1 | 37 | 29 | 1 | | 1 | 1 |
| 8 | 2 | 58 | 25 | 1 | 1 | 1 | 1 |
| 8 | 2 | 22 | 29 | 1 | | 1 | 1 |
| 8 | 1 | 51 | 19 | 1 | | 1 | 0 |
| 8 | 2 | 24 | 31 | 1 | | 1 | 0 |
| 8 | 2 | 25 | 23 | 1 | | 1 | 1 |
| 8 | 1 | 44 | 19 | 1 | 3 | 1 | 0 |
| 8 | 2 | 33 | 26 | 1 | - | 1 | 0 |
| 8 | 2 | 23 | 19 | 1 | | 1 | 0 |
| 8 | 2 | 49 | 29 | 1 | | 1 | 0 |
| 8 | 2 | 23 | 23 | 1 | | 1 | 1 |
| 8 | 2 | 43 | 24 | 1 | | 1 | 1 |
| 8 | 2 | 79 | 32 | 1 | | 1 | 0 |
| 8 | 2 | 40 | 22 | 1 | | 1 | 1 |
| 8 | 2 | 55 | 28 | 1 | | 1 | 1 |
| 8 | 2 | 24 | 21 | 1 | | 1 | 0 |
| 8 | 2 | 28 | 21 | 1 | | 2 | 1 |
| 8 | 1 | 55 | 22 | 1 | | 1 | 0 |
| 8 | 2 | 18 | 27 | 1 | | 1 | 0 |
| 8 | 2 | 21 | 18 | 1 | | 1 | 0 |
| 8 | 1 | 29 | 27 | 1 | | 1 | 0 |
| 8 | 1 | 53 | 31 | 1 | | 1 | 0 |

| | | | | | | | |
|---|---|---|---|---|---|---|---|
| 8 | 1 | 53 | 25 | 1 | | 1 | 1 |
| 8 | 1 | 25 | 26 | 1 | 1 | 1 | 0 |
| 8 | 1 | 56 | 23 | 1 | | 1 | 0 |
| 8 | 1 | 24 | 25 | 1 | | 1 | 0 |
| 8 | 1 | 59 | 26 | 1 | 3 | 1 | 1 |
| 8 | 1 | 74 | 21 | 1 | | 1 | 0 |
| 8 | 2 | 80 | 22 | 1 | | 1 | 0 |
| 8 | 1 | 62 | 21 | 1 | | 1 | 0 |
| 8 | 1 | 26 | 23 | 1 | 1 | 1 | 0 |
| 8 | 2 | 27 | 22 | 1 | | 1 | 1 |
| 8 | 2 | 43 | 27 | 1 | | 1 | 0 |
| 8 | 2 | 51 | 24 | 1 | 3 | 1 | 0 |
| 8 | 1 | 53 | 26 | 1 | | 1 | 0 |
| 8 | 1 | 54 | 31 | 1 | 1 | 1 | 0 |
| 8 | 1 | 71 | 28 | 1 | | 1 | 0 |
| 8 | 1 | 66 | 19 | 1 | | 1 | 1 |
| 8 | 2 | 71 | 26 | 1 | | 1 | 1 |
| 8 | 2 | 23 | 23 | 1 | | 1 | 1 |
| 8 | 2 | 61 | 20 | 1 | | 1 | 0 |
| 8 | 2 | 30 | 21 | 1 | | 1 | 0 |
| 8 | 1 | 58 | 22 | 1 | | 1 | 0 |
| 8 | 2 | 46 | 18 | 1 | | 1 | 1 |
| 8 | 1 | 41 | 24 | 1 | | 1 | 1 |
| 8 | 2 | 35 | 25 | 1 | | 1 | 1 |
| 8 | 1 | 70 | 26 | 1 | | 1 | 1 |
| 8 | 2 | 77 | 27 | 1 | | 1 | 1 |
| 8 | 1 | 88 | 17 | 1 | | 1 | 0 |
| 8 | 1 | 66 | 26 | 1 | | 1 | 0 |
| 8 | 1 | 33 | 25 | 1 | | 1 | 1 |
| 8 | 1 | 65 | 31 | 1 | | 1 | 1 |
| 8 | 1 | 68 | 26 | 1 | | 1 | 0 |
| 8 | 1 | 45 | 24 | 1 | | 1 | 1 |
| 8 | 2 | 55 | 25 | 1 | 2 | 1 | 0 |
| 8 | 1 | 62 | 26 | 1 | -2 | 1 | 1 |
| 8 | 2 | 21 | 25 | 1 | | 1 | 1 |
| 8 | 1 | 53 | 30 | 1 | | 1 | 1 |
| 8 | 2 | 39 | 31 | 1 | | 1 | 1 |
| 8 | 2 | 21 | 28 | 1 | | 1 | 0 |
| 8 | 2 | 30 | 27 | 1 | 3 | 1 | 1 |
| 8 | 1 | 41 | 18 | 1 | | 1 | 1 |
| 8 | 2 | 56 | 21 | 1 | 2 | 1 | 1 |
| 8 | 1 | 61 | 24 | 1 | | 1 | 1 |
| 8 | 1 | 66 | 25 | 1 | | 1 | 0 |
| 8 | 1 | 48 | 24 | 1 | 1 | 1 | 1 |
| 8 | 1 | 27 | 23 | 1 | 5 | 1 | 1 |
| 8 | 2 | 29 | 28 | 1 | 5 | 1 | 1 |
| 8 | 1 | 52 | 19 | 1 | | 1 | 1 |
| 8 | 1 | 27 | 28 | 1 | | 1 | 1 |
| 8 | 1 | 25 | 22 | 1 | 5 | 1 | 0 |
| 8 | 1 | 85 | 24 | 1 | 4 | 1 | 0 |

| | | | | | | | |
|---|---|---|---|---|---|---|---|
| 8 | 2 | 65 | 26 | 1 | | 2 | 1 |
| 8 | 1 | 56 | 20 | 1 | | 1 | 1 |
| 8 | 2 | 34 | 28 | 1 | | 1 | 1 |
| 8 | 1 | 31 | 32 | 1 | | 1 | 1 |
| 8 | 1 | 49 | 20 | 1 | - | 1 | 0 |
| 8 | 2 | 26 | 27 | 1 | | 1 | 1 |
| 8 | 2 | 25 | 25 | 1 | | 1 | 1 |
| 8 | 1 | 44 | 31 | 1 | | 1 | 0 |
| 8 | 2 | 15 | 32 | 1 | | 1 | 0 |
| 8 | 2 | 58 | 21 | 1 | | 1 | 0 |
| 8 | 2 | 63 | 28 | 1 | | 1 | 0 |
| 8 | 2 | 30 | 23 | 1 | | 1 | 1 |
| 8 | 2 | 46 | 29 | 1 | | 1 | 1 |
| 8 | 2 | 87 | 22 | 1 | | 1 | 0 |
| 8 | 2 | 70 | 23 | 1 | 1 | 1 | 1 |
| 8 | 2 | 30 | 24 | 1 | 1 | 1 | 1 |
| 8 | 1 | 69 | 32 | 1 | 1 | 1 | 1 |
| 8 | 1 | 36 | 31 | 1 | | 2 | 1 |
| 8 | 2 | 28 | 26 | 1 | 1 | 1 | 1 |
| 8 | 1 | 45 | 30 | 1 | | 1 | 0 |
| 8 | 1 | 33 | 32 | 1 | | 1 | 1 |
| 8 | 2 | 61 | 28 | 1 | | 1 | 0 |
| 8 | 1 | 77 | 22 | 1 | | 2 | 1 |
| 8 | 2 | 69 | 30 | 1 | | 2 | 1 |
| 8 | 1 | 51 | 23 | 1 | | 1 | 0 |
| 8 | 1 | 35 | 26 | 1 | | 1 | 0 |
| 8 | 2 | 33 | 31 | 1 | | 1 | 0 |
| 8 | 1 | 52 | 23 | 1 | | 1 | 1 |
| 8 | 1 | 24 | 28 | 1 | | 1 | 0 |
| 8 | 1 | 36 | 32 | 1 | | 1 | 0 |
| 8 | 2 | 61 | 25 | 1 | | 1 | 1 |
| 8 | 2 | 79 | 20 | 1 | | 1 | 2 |
| 8 | 1 | 70 | 20 | 1 | | 1 | 2 |
| 8 | 2 | 44 | 24 | 1 | | 1 | 1 |
| 8 | 2 | 16 | 25 | 1 | | 1 | 0 |
| 8 | 1 | 40 | 30 | 1 | | 1 | 0 |
| 8 | 1 | 43 | 29 | 1 | 14 | 1 | 1 |
| 8 | 2 | 29 | 22 | 1 | 1 | 1 | 1 |
| 8 | 1 | 30 | 28 | 1 | | 1 | 1 |
| 8 | 2 | 37 | 29 | 1 | | 1 | 1 |
| 8 | 1 | 52 | 27 | 1 | 1 | 1 | 1 |
| 8 | 2 | 46 | 30 | 1 | | 1 | 1 |
| 8 | 2 | 61 | 29 | 1 | | 1 | 0 |
| 8 | 1 | 39 | 21 | 1 | | 1 | 0 |
| 8 | 2 | 39 | 29 | 1 | 4 | 1 | 0 |
| 8 | 1 | 24 | 17 | 1 | 2 | 1 | 0 |
| 8 | 2 | 36 | 28 | 1 | | 1 | 0 |
| 8 | 1 | 89 | 26 | 1 | | 1 | 0 |
| 8 | 1 | 24 | 20 | 1 | | 1 | 1 |
| 8 | 1 | 30 | 24 | 1 | 1 | 1 | 0 |

| | | | | | | | |
|---|---|---|---|---|---|---|---|
| 8 | 2 | 30 | 26 | 1 | 1 | 1 | 1 |
| 8 | 1 | 28 | 26 | 1 | 2 | 1 | 1 |
| 8 | 2 | 23 | 30 | 1 | 2 | 1 | 1 |
| 8 | 1 | 46 | 22 | 1 | | 1 | 1 |
| 8 | 1 | 59 | 30 | 1 | | 1 | 1 |
| 8 | 1 | 68 | 29 | 1 | 2 | 1 | 0 |
| 8 | 1 | 36 | 24 | 1 | | 1 | 1 |
| 8 | 1 | 22 | 22 | 1 | | 1 | 0 |
| 8 | 2 | 53 | 18 | 1 | | 1 | 1 |
| 8 | 2 | 59 | 29 | 1 | 2 | 1 | 1 |
| 8 | 2 | 29 | 22 | 1 | 5 | 1 | 1 |
| 8 | 2 | 60 | 23 | 1 | 8 | 1 | 1 |
| 8 | 1 | 66 | 27 | 1 | | 1 | 1 |
| 8 | 1 | 59 | 23 | 1 | | 2 | 0 |
| 8 | 1 | 55 | 30 | 1 | | 1 | 1 |
| 8 | 2 | 51 | 20 | 1 | 4 | 1 | 1 |
| 8 | 1 | 60 | 22 | 1 | - | 1 | 1 |
| 8 | 2 | 32 | 32 | 1 | | 1 | 0 |
| 8 | 2 | 45 | 25 | 1 | | 1 | 1 |
| 8 | 2 | 29 | 27 | 1 | | 1 | 1 |
| 8 | 2 | 31 | 26 | 1 | 4 | 1 | 1 |
| 8 | 2 | 34 | 27 | 1 | 1 | 1 | 1 |
| 8 | 1 | 36 | 31 | 1 | | 1 | 0 |
| 8 | 1 | 35 | 31 | 1 | 3 | 1 | 1 |
| 8 | 2 | 50 | 25 | 1 | 1 | 1 | 0 |
| 8 | 2 | 42 | 29 | 1 | 2 | 1 | 0 |
| 8 | 1 | 51 | 31 | 1 | 7 | 1 | 0 |
| 8 | 1 | 19 | 33 | 1 | | 1 | 0 |
| 8 | 2 | 35 | 28 | 1 | 1 | 1 | 1 |
| 8 | 1 | 68 | 26 | 1 | | 1 | 0 |
| 8 | 2 | 34 | 29 | 1 | | 1 | 0 |
| 8 | 1 | 55 | 34 | 1 | | 1 | 0 |
| 8 | 1 | 24 | 28 | 1 | | 1 | 1 |
| 8 | 2 | 47 | 23 | 1 | | 1 | 0 |
| 8 | 1 | 46 | 23 | 1 | | 1 | 0 |
| 8 | 1 | 54 | 25 | 1 | | 1 | 0 |
| 8 | 2 | 18 | 27 | 1 | | 1 | 0 |
| 8 | 1 | 10 | 28 | 1 | | 1 | 1 |
| 8 | 1 | 44 | 28 | 1 | | 1 | 1 |
| 8 | 1 | 42 | 31 | 1 | | 1 | 0 |
| 8 | 1 | 13 | 24 | 1 | -1 | 1 | 1 |
| 8 | 2 | 62 | 30 | 1 | | 1 | 1 |
| 8 | 1 | 58 | 19 | 1 | 1 | 1 | 0 |
| 8 | 2 | 29 | 18 | 1 | | 1 | 1 |
| 8 | 1 | 47 | 26 | 1 | | 1 | 0 |
| 8 | 2 | 23 | 32 | 1 | | 1 | 0 |
| 8 | 2 | 35 | 24 | 1 | | 1 | 1 |
| 8 | 2 | 27 | 26 | 1 | 1 | 1 | 0 |
| 8 | 1 | 58 | 26 | 1 | 1 | 1 | 0 |
| 8 | 1 | 61 | 26 | 1 | - | 1 | 0 |

| | | | | | | | |
|---|---|---|---|---|---|---|---|
| 8 | 1 | 35 | 28 | 1 | 2 | 1 | 1 |
| 8 | 2 | 65 | 27 | 1 | 3 | 1 | 1 |
| 8 | 2 | 55 | 17 | 1 | | 1 | 1 |
| 8 | 1 | 22 | 30 | 1 | | 1 | 1 |
| 8 | 2 | 43 | 22 | 1 | | 1 | 0 |
| 8 | 1 | 66 | 30 | 1 | | 1 | 1 |
| 8 | 1 | 87 | 20 | 1 | 3 | 1 | 0 |
| 8 | 1 | 62 | 25 | 1 | | 1 | 0 |
| 8 | 2 | 64 | 30 | 1 | | 1 | 0 |
| 8 | 1 | 38 | 26 | 1 | | 1 | 1 |
| 8 | 2 | 60 | 22 | 1 | | 1 | 0 |
| 8 | 1 | 57 | 29 | 1 | | 1 | 0 |
| 8 | 2 | 1 | 20 | 1 | | 1 | 1 |
| 8 | 2 | 57 | 21 | 1 | | 1 | 1 |
| 8 | 1 | 31 | 32 | 1 | | 1 | 1 |
| 8 | 1 | 27 | 23 | 1 | 8 | 1 | 1 |
| 8 | 2 | 27 | 22 | 1 | | 1 | 1 |
| 8 | 1 | 48 | 29 | 1 | | 1 | 1 |
| 8 | 1 | 48 | 26 | 1 | | 1 | 0 |
| 8 | 2 | 23 | 32 | 1 | 3 | 1 | 1 |
| 8 | 2 | 58 | 25 | 1 | | 1 | 1 |
| 8 | 2 | 20 | 26 | 1 | | 1 | 1 |
| 8 | 2 | 86 | 28 | 1 | - | 1 | 0 |
| 8 | 2 | 59 | 30 | 1 | 1 | 1 | 0 |
| 8 | 2 | 43 | 24 | 1 | 2 | 1 | 1 |
| 8 | 1 | 27 | 26 | 1 | | 1 | 1 |
| 8 | 1 | 56 | 18 | 1 | 5 | 1 | 0 |
| 8 | 2 | 75 | 28 | 1 | | 1 | 0 |
| 8 | 1 | 19 | 21 | 1 | | 1 | 0 |
| 8 | 2 | 64 | 20 | 1 | | 1 | 1 |
| 8 | 2 | 41 | 25 | 1 | | 1 | 1 |
| 8 | 2 | 29 | 25 | 1 | 1 | 1 | 0 |
| 8 | 1 | 51 | 23 | 1 | 2 | 1 | 0 |
| 8 | 2 | 3 | 26 | 1 | 2 | 1 | 0 |
| 8 | 2 | 47 | 26 | 1 | | 1 | 0 |
| 8 | 2 | 20 | 26 | 1 | | 1 | 1 |
| 8 | 2 | 21 | 22 | 1 | 1 | 1 | 1 |
| 8 | 1 | 43 | 20 | 1 | | 1 | 0 |
| 8 | 1 | 30 | 32 | 1 | | 1 | 0 |
| 8 | 1 | 78 | 27 | 1 | | 2 | 1 |
| 8 | 2 | 65 | 32 | 1 | | 2 | 1 |
| 8 | 1 | 64 | 26 | 1 | | 1 | 0 |
| 8 | 1 | 54 | 26 | 1 | | 1 | 0 |
| 8 | 2 | 57 | 28 | 1 | | 1 | 0 |
| 8 | 2 | 43 | 32 | 1 | | 1 | 0 |
| 8 | 1 | 35 | 25 | 1 | | 1 | 1 |
| 8 | 1 | 48 | 29 | 1 | | 2 | 1 |
| 8 | 2 | 59 | 26 | 1 | | 2 | 0 |
| 8 | 1 | 54 | 23 | 1 | | 1 | 0 |
| 8 | 1 | 76 | 26 | 1 | | 2 | 0 |

| | | | | | | | |
|---|---|---|---|---|---|---|---|
| 8 | 1 | 24 | 31 | 1 | 1 | 1 | 0 |
| 8 | 2 | 63 | 22 | 1 | 1 | 1 | 1 |
| 8 | 2 | 24 | 28 | 1 | | 1 | 1 |
| 8 | 2 | 57 | 31 | 1 | | 1 | 1 |
| 8 | 2 | 72 | 19 | 1 | | 2 | 1 |
| 8 | 1 | 44 | 20 | 1 | | 1 | 0 |
| 8 | 1 | 57 | 22 | 1 | | 1 | 1 |
| 8 | 2 | 16 | 21 | 1 | 0 | 1 | 1 |
| 8 | 2 | 31 | 21 | 1 | | 1 | 1 |
| 8 | 2 | 66 | 24 | 1 | | 1 | 1 |
| 8 | 1 | 59 | 29 | 1 | | 1 | 0 |
| 8 | 2 | 63 | 29 | 1 | | 1 | 0 |
| 8 | 2 | 26 | 31 | 1 | | 1 | 0 |
| 8 | 1 | 47 | 24 | 1 | 4 | 1 | 1 |
| 8 | 2 | 32 | 20 | 1 | 1 | 1 | 0 |
| 8 | 1 | 47 | 25 | 1 | 1 | 1 | 0 |
| 8 | 1 | 60 | 24 | 1 | 4 | 1 | 0 |
| 8 | 2 | 40 | 28 | 1 | 5 | 1 | 0 |
| 8 | 2 | 22 | 23 | 1 | | 1 | 0 |
| 8 | 2 | 33 | 25 | 1 | | 1 | 0 |
| 8 | 1 | 82 | 26 | 1 | | 2 | 0 |
| 8 | 1 | 21 | 25 | 1 | 2 | 1 | 0 |
| 8 | 2 | 47 | 27 | 1 | 14 | 1 | 0 |
| 8 | 2 | 35 | 28 | 1 | | 1 | 1 |
| 8 | 1 | 8 | 20 | 1 | 2 | 1 | 0 |
| 8 | 2 | 12 | 32 | 1 | 4 | 1 | 0 |
| 8 | 2 | 11 | 24 | 1 | 4 | 1 | 1 |
| 8 | 2 | 50 | 27 | 1 | | 1 | 0 |
| 8 | 1 | 69 | 28 | 1 | 8 | 1 | 0 |
| 8 | 1 | 16 | 29 | 1 | | 1 | 1 |
| 8 | 1 | 47 | 23 | 1 | | 1 | 0 |
| 8 | 1 | 81 | 22 | 1 | 4 | 1 | 1 |
| 8 | 2 | 81 | 26 | 1 | 4 | 1 | 1 |
| 8 | 2 | 56 | 21 | 1 | | 1 | 0 |
| 8 | 2 | 87 | 32 | 1 | | 1 | 1 |
| 8 | 2 | 15 | 30 | 1 | | 1 | 1 |
| 8 | 1 | 39 | 28 | 1 | | 1 | 0 |
| 8 | 1 | 33 | 18 | 1 | | 1 | 0 |
| 8 | 2 | 38 | 28 | 1 | | 1 | 0 |
| 8 | 2 | 28 | 23 | 1 | | 1 | 0 |
| 8 | 1 | 43 | 30 | 1 | | 1 | 0 |
| 8 | 2 | 24 | 25 | 1 | | 1 | 0 |
| 8 | 2 | 61 | 30 | 1 | | 2 | 0 |
| 8 | 1 | 87 | 28 | 1 | | 1 | 1 |
| 8 | 1 | 58 | 24 | 1 | | 1 | 1 |
| 8 | 1 | 38 | 16 | 1 | | 1 | 1 |
| 8 | 2 | 55 | 20 | 1 | | 1 | 1 |
| 8 | 2 | 45 | 17 | 1 | | 1 | 0 |
| 8 | 2 | 8 | 22 | 1 | | 1 | 1 |
| 8 | 2 | 59 | 23 | 1 | | 1 | 1 |

| | | | | | | | |
|---|---|---|---|---|---|---|---|
| 8 | 2 | 4 | 25 | 1 | | 1 | 1 |
| 8 | 2 | 53 | 20 | 1 | 0 | 1 | 0 |
| 8 | 1 | 48 | 21 | 1 | 7 | 1 | 0 |
| 8 | 1 | 40 | 28 | 1 | | 1 | 0 |
| 8 | 2 | 19 | 17 | 1 | | 1 | 1 |
| 8 | 1 | 20 | 19 | 1 | | 1 | 1 |
| 8 | 1 | 40 | 23 | 1 | | 1 | 1 |
| 8 | 1 | 55 | 25 | 1 | | 1 | 1 |
| 8 | 1 | 53 | 22 | 1 | 2 | 1 | 1 |
| 8 | 2 | 53 | 22 | 1 | 6 | 1 | 1 |
| 8 | 2 | 27 | 20 | 1 | | 1 | 1 |
| 8 | 2 | 60 | 28 | 1 | | 1 | 1 |
| 8 | 2 | 53 | 26 | 1 | 1 | 1 | 1 |
| 8 | 1 | 31 | 28 | 1 | - | 1 | 1 |
| 8 | 1 | 23 | 30 | 1 | | 1 | 0 |
| 8 | 2 | 46 | 21 | 1 | | 1 | 1 |
| 8 | 2 | 25 | 28 | 1 | 2 | 1 | 1 |
| 8 | 2 | 73 | 29 | 1 | | 2 | 1 |
| 8 | 2 | 58 | 19 | 1 | 5 | 1 | 1 |
| 8 | 2 | 37 | 33 | 1 | | 1 | 0 |
| 9 | 2 | 61 | 23 | 1 | 2 | 1 | 0 |
| 9 | 1 | 76 | 20 | 1 | | 1 | 0 |
| 9 | 1 | 57 | 24 | 1 | | 1 | 0 |
| 9 | 1 | 73 | 27 | 1 | | 1 | 0 |
| 9 | 1 | 34 | 24 | 1 | | 1 | 1 |
| 9 | 1 | 70 | 24 | 1 | | 1 | 0 |
| 10 | 1 | 28 | 24 | 1 | | 1 | 0 |
| 10 | 2 | 47 | 26 | 1 | | 1 | 1 |
| 10 | 1 | 23 | 25 | 1 | 2 | 1 | 1 |
| 10 | 1 | 51 | 26 | 1 | 5 | 1 | 1 |
| 10 | 1 | 84 | 23 | 1 | | 1 | 1 |
| 10 | 1 | 33 | 27 | 1 | | 1 | 0 |
| 10 | 1 | 63 | 28 | 1 | | 1 | 0 |
| 10 | 1 | 26 | 32 | 1 | | 1 | 0 |
| 10 | 1 | 39 | 30 | 1 | | 1 | 0 |
| 10 | 2 | 59 | 21 | 1 | | 1 | 1 |
| 10 | 2 | 16 | 28 | 1 | 5 | 1 | 1 |
| 10 | 2 | 49 | 23 | 1 | | 1 | 1 |
| 10 | 1 | 53 | 26 | 1 | | 1 | 1 |
| 10 | 1 | 7 | 21 | 1 | 1 | 1 | 1 |
| 10 | 2 | 59 | 24 | 1 | 2 | 1 | 1 |
| 10 | 2 | 20 | 19 | 1 | | 1 | 1 |
| 10 | 2 | 42 | 20 | 1 | | 1 | 1 |
| 10 | 1 | 73 | 21 | 1 | | 1 | 1 |
| 10 | 2 | 56 | 21 | 1 | | 1 | 1 |
| 10 | 1 | 55 | 23 | 1 | | 1 | 1 |
| 10 | 2 | 55 | 23 | 1 | | 1 | 1 |
| 10 | 1 | 2 | 24 | 1 | | 1 | 1 |
| 10 | 1 | 3 | 25 | 1 | | 1 | 1 |
| 10 | 1 | 47 | 24 | 1 | 2 | 1 | 1 |

| | | | | | | | |
|---|---|---|---|---|---|---|---|
| 10 | 1 | 51 | 22 | 1 | | 1 | 1 |
| 10 | 2 | 58 | 26 | 1 | 1 | 1 | 1 |
| 10 | 2 | 34 | 25 | 1 | | 1 | 1 |
| 10 | 2 | 46 | 25 | 1 | -1 | 1 | 1 |
| 10 | 1 | 29 | 30 | 1 | | 1 | 1 |
| 10 | 1 | 60 | 25 | 1 | 3 | 1 | 1 |
| 10 | 2 | 11 | 24 | 1 | 6 | 1 | 1 |
| 10 | 1 | 14 | 18 | 1 | | 1 | 1 |
| 10 | 1 | 5 | 22 | 1 | | 1 | 1 |
| 10 | 2 | 60 | 24 | 1 | | 1 | 1 |
| 10 | 2 | 31 | 27 | 1 | | 1 | 1 |
| 10 | 2 | 70 | 32 | 1 | | 1 | 1 |
| 10 | 2 | 19 | 21 | 1 | | 1 | 1 |
| 10 | 1 | 38 | 29 | 1 | | 1 | 1 |
| 10 | 2 | 66 | 23 | 1 | | 1 | 1 |
| 10 | 2 | 31 | 23 | 1 | | 1 | 1 |
| 10 | 2 | 27 | 28 | 1 | | 1 | 1 |
| 10 | 1 | 35 | 24 | 1 | | 1 | 0 |
| 10 | 2 | 30 | 22 | 1 | | 1 | 1 |
| 10 | 1 | 21 | 22 | 1 | 2 | 1 | 1 |
| 10 | 1 | 50 | 26 | 1 | 2 | 1 | 1 |
| 10 | 2 | 54 | 25 | 1 | 2 | 1 | 0 |
| 10 | 2 | 82 | 25 | 1 | | 1 | 0 |
| 10 | 2 | 44 | 24 | 1 | 2 | 1 | 1 |
| 10 | 2 | 56 | 25 | 1 | | 1 | 0 |
| 10 | 2 | 24 | 24 | 1 | | 1 | 1 |
| 10 | 1 | 22 | 28 | 1 | 4 | 1 | 1 |
| 10 | 2 | 58 | 23 | 1 | | 1 | 1 |
| 10 | 1 | 42 | 22 | 1 | | 1 | 1 |
| 10 | 1 | 20 | 28 | 1 | | 1 | 1 |
| 10 | 2 | 19 | 26 | 1 | | 1 | 2 |
| 10 | 2 | 55 | 25 | 1 | | 1 | 1 |
| 10 | 1 | 32 | 25 | 1 | | 1 | 1 |
| 10 | 1 | 37 | 26 | 1 | | 1 | 1 |
| 10 | 2 | 44 | 22 | 1 | | 1 | 0 |
| 10 | 1 | 65 | 24 | 1 | 4 | 1 | 1 |
| 10 | 2 | 43 | 20 | 1 | | 1 | 1 |
| 10 | 2 | 48 | 27 | 1 | | 1 | 1 |
| 10 | 2 | 19 | 27 | 1 | | 1 | 1 |
| 10 | 2 | 40 | 27 | 1 | | 1 | 1 |
| 10 | 1 | 42 | 28 | 1 | | 1 | 1 |
| 10 | 2 | 18 | 22 | 1 | | 1 | 0 |
| 10 | 1 | 41 | 27 | 1 | | 1 | 1 |
| 10 | 1 | 27 | 28 | 1 | | 1 | 1 |
| 10 | 1 | 52 | 20 | 1 | 2 | 1 | 1 |
| 10 | 2 | 58 | 24 | 1 | 2 | 1 | 1 |
| 10 | 2 | 44 | 28 | 1 | 1 | 1 | 1 |
| 10 | 2 | 36 | 24 | 1 | 2 | 1 | 1 |
| 10 | 2 | 62 | 27 | 1 | 2 | 1 | 1 |
| 10 | 2 | 52 | 26 | 1 | | 1 | 2 |

| | | | | | | | |
|---|---|---|---|---|---|---|---|
| 10 | 1 | 54 | 25 | 1 | 3 | 1 | 1 |
| 10 | 2 | 17 | 26 | 1 | | 1 | 0 |
| 10 | 1 | 45 | 24 | 1 | | 1 | 1 |
| 10 | 1 | 40 | 29 | 1 | | 1 | 1 |
| 10 | 2 | 47 | 25 | 1 | | 1 | 0 |
| 10 | 1 | 41 | 30 | 1 | | 1 | 0 |
| 10 | 2 | 46 | 31 | 1 | 1 | 1 | 1 |
| 10 | 2 | 57 | 18 | 1 | 2 | 1 | 1 |
| 10 | 2 | 10 | 27 | 1 | | 1 | 0 |
| 10 | 2 | 47 | 21 | 1 | | 1 | 1 |
| 10 | 1 | 47 | 30 | 1 | | 1 | 1 |
| 10 | 1 | 42 | 26 | 1 | | 1 | 0 |
| 10 | 2 | 61 | 23 | 1 | | 1 | 1 |
| 10 | 2 | 37 | 28 | 1 | | 1 | 1 |
| 10 | 2 | 59 | 31 | 1 | | 1 | 1 |
| 10 | 1 | 63 | 27 | 1 | 0 | 1 | 1 |
| 10 | 1 | 52 | 25 | 1 | | 1 | 1 |
| 10 | 1 | 58 | 30 | 1 | | 1 | 1 |
| 10 | 1 | 20 | 32 | 1 | | 1 | 1 |
| 10 | 2 | 25 | 24 | 1 | | 1 | 0 |
| 10 | 2 | 64 | 23 | 1 | | 1 | 1 |
| 10 | 1 | 20 | 25 | 1 | | 1 | 1 |
| 10 | 2 | 35 | 27 | 1 | | 1 | 1 |
| 10 | 1 | 88 | 25 | 1 | | 1 | 0 |
| 10 | 2 | 53 | 25 | 1 | | 1 | 1 |
| 10 | 1 | 39 | 24 | 1 | 1 | 1 | 0 |
| 10 | 2 | 21 | 22 | 1 | 2 | 1 | 0 |
| 10 | 1 | 1 | 32 | 1 | | 1 | 1 |
| 10 | 1 | 40 | 29 | 1 | | 1 | 0 |
| 10 | 2 | 15 | 22 | 1 | 4 | 1 | 1 |
| 10 | 2 | 39 | 25 | 1 | -2 | 1 | 1 |
| 10 | 2 | 54 | 19 | 1 | 3 | 1 | 1 |
| 10 | 1 | 47 | 24 | 1 | | 1 | 1 |
| 10 | 1 | 23 | 25 | 1 | | 1 | 1 |
| 10 | 1 | 63 | 28 | 1 | | 1 | 1 |
| 10 | 2 | 52 | 31 | 1 | | 1 | 1 |
| 10 | 1 | 60 | 26 | 1 | | 1 | 0 |
| 10 | 2 | 69 | 25 | 1 | | 2 | 1 |
| 10 | 1 | 24 | 21 | 1 | | 1 | 1 |
| 10 | 1 | 36 | 24 | 1 | | 1 | 1 |
| 10 | 2 | 37 | 25 | 1 | | 1 | 1 |
| 10 | 1 | 9 | 26 | 1 | | 1 | 1 |
| 10 | 2 | 41 | 27 | 1 | | 1 | 1 |
| 10 | 2 | 58 | 30 | 1 | | 1 | 1 |
| 10 | 1 | 51 | 32 | 1 | | 1 | 1 |
| 10 | 1 | 15 | 31 | 1 | | 1 | 1 |
| 10 | 1 | 31 | 23 | 1 | | 1 | 1 |
| 10 | 1 | 57 | 25 | 1 | 3 | 1 | 1 |
| 10 | 2 | 59 | 17 | 1 | | 1 | 1 |
| 10 | 1 | 70 | 26 | 1 | 1 | 1 | 1 |

| | | | | | | | |
|---|---|---|---|---|---|---|---|
| 10 | 1 | 54 | 22 | 1 | | 1 | 1 |
| 10 | 1 | 25 | 22 | 1 | | 1 | 0 |
| 10 | 1 | 59 | 31 | 1 | | 1 | 0 |
| 10 | 2 | 60 | 25 | 1 | | 1 | 1 |
| 10 | 2 | 41 | 27 | 1 | 5 | 1 | 1 |
| 10 | 2 | 34 | 27 | 1 | 5 | 1 | 0 |
| 10 | 2 | 39 | 25 | 1 | | 1 | 0 |
| 10 | 1 | 31 | 30 | 1 | 6 | 1 | 1 |
| 10 | 1 | 19 | 27 | 1 | | 1 | 1 |
| 10 | 2 | 55 | 31 | 1 | | 1 | 1 |
| 10 | 2 | 52 | 26 | 1 | 1 | 1 | 0 |
| 10 | 1 | 30 | 24 | 1 | 3 | 1 | 0 |
| 10 | 1 | 38 | 23 | 1 | | 1 | 0 |
| 10 | 1 | 3 | 30 | 1 | 1 | 1 | 1 |
| 10 | 2 | 48 | 21 | 1 | 3 | 1 | 1 |
| 10 | 1 | 19 | 22 | 1 | | 1 | 1 |
| 10 | 1 | 32 | 23 | 1 | | 1 | 1 |
| 10 | 1 | 28 | 23 | 1 | | 1 | 1 |
| 10 | 1 | 23 | 24 | 1 | | 1 | 1 |
| 10 | 2 | 49 | 30 | 1 | | 1 | 1 |
| 10 | 1 | 52 | 28 | 1 | 2 | 1 | 1 |
| 10 | 1 | 49 | 29 | 1 | | 1 | 1 |
| 10 | 2 | 44 | 22 | 1 | | 1 | 0 |
| 10 | 2 | 58 | 28 | 1 | | 1 | 1 |
| 10 | 2 | 41 | 30 | 1 | | 1 | 0 |
| 10 | 2 | 78 | 24 | 1 | 2 | 1 | 1 |
| 10 | 2 | 44 | 24 | 1 | | 1 | 1 |
| 10 | 2 | 62 | 28 | 1 | | 1 | 1 |
| 10 | 2 | 32 | 25 | 1 | | 1 | 0 |
| 10 | 2 | 25 | 18 | 1 | | 1 | 1 |
| 10 | 2 | 66 | 30 | 1 | | 2 | 1 |
| 10 | 1 | 25 | 28 | 1 | | 1 | 0 |
| 10 | 1 | 82 | 30 | 1 | | 2 | 1 |
| 10 | 2 | 46 | 23 | 1 | | 1 | 0 |
| 10 | 2 | 20 | 24 | 1 | | 1 | 0 |
| 10 | 1 | 90 | 29 | 1 | | 1 | 0 |
| 10 | 2 | 61 | 30 | 1 | | 1 | 0 |
| 10 | 2 | 56 | 25 | 1 | | 1 | 0 |
| 10 | 1 | 8 | 29 | 1 | | 1 | 0 |
| 10 | 1 | 33 | 26 | 1 | | 1 | 1 |
| 10 | 2 | 35 | 19 | 1 | 1 | 1 | 1 |
| 10 | 2 | 55 | 22 | 1 | | 1 | 1 |
| 10 | 2 | 21 | 31 | 1 | | 1 | 1 |
| 10 | 1 | 21 | 32 | 1 | | 1 | 0 |
| 10 | 1 | 54 | 25 | 1 | 2 | 1 | 1 |
| 10 | 2 | 33 | 26 | 1 | | 1 | 1 |
| 10 | 2 | 6 | 24 | 1 | 1 | 1 | 1 |
| 10 | 1 | 38 | 30 | 1 | | 1 | 1 |
| 10 | 2 | 54 | 23 | 1 | 1 | 1 | 1 |
| 10 | 2 | 43 | 22 | 1 | | 1 | 1 |

| | | | | | | | |
|---|---|---|---|---|---|---|---|
| 10 | 2 | 24 | 21 | 1 | | 1 | 1 |
| 10 | 2 | 39 | 22 | 1 | | 1 | 0 |
| 10 | 1 | 82 | 30 | 1 | 4 | 1 | 1 |
| 10 | 1 | 59 | 32 | 1 | | 1 | 1 |
| 10 | 1 | 31 | 24 | 1 | | 1 | 1 |
| 10 | 2 | 73 | 30 | 1 | | 1 | 1 |
| 10 | 1 | 12 | 23 | 1 | | 1 | 1 |
| 10 | 2 | 28 | 26 | 1 | 2 | 1 | 0 |
| 10 | 2 | 54 | 28 | 1 | 3 | 1 | 0 |
| 10 | 1 | 53 | 28 | 1 | 3 | 1 | 0 |
| 10 | 2 | 27 | 20 | 1 | 1 | 1 | 1 |
| 10 | 2 | 69 | 22 | 1 | | 1 | 1 |
| 10 | 1 | 10 | 26 | 1 | | 1 | 1 |
| 10 | 1 | 33 | 27 | 1 | | 1 | 1 |
| 10 | 2 | 22 | 28 | 1 | | 1 | 1 |
| 10 | 1 | 37 | 31 | 1 | | 1 | 1 |
| 10 | 2 | 29 | 32 | 1 | | 1 | 1 |
| 10 | 1 | 15 | 22 | 1 | 1 | 1 | 2 |
| 10 | 2 | 25 | 19 | 1 | | 1 | 2 |
| 10 | 1 | 81 | 31 | 1 | | 1 | 2 |
| 10 | 2 | 29 | 19 | 1 | 1 | 1 | 1 |
| 10 | 1 | 58 | 24 | 1 | | 1 | 1 |
| 10 | 1 | 14 | 26 | 1 | | 1 | 1 |
| 10 | 1 | 26 | 32 | 1 | | 1 | 0 |
| 10 | 1 | 50 | 22 | 1 | 3 | 1 | 1 |
| 10 | 1 | 43 | 23 | 1 | | 1 | 1 |
| 10 | 2 | 31 | 30 | 1 | | 1 | 1 |
| 10 | 1 | 48 | 28 | 1 | 1 | 1 | 1 |
| 10 | 1 | 45 | 29 | 1 | | 1 | 0 |
| 10 | 1 | 31 | 21 | 1 | | 1 | 1 |
| 10 | 2 | 39 | 22 | 1 | | 1 | 1 |
| 10 | 1 | 37 | 22 | 1 | | 1 | 1 |
| 10 | 2 | 60 | 25 | 1 | | 1 | 1 |
| 10 | 1 | 37 | 27 | 1 | | 1 | 1 |
| 10 | 1 | 21 | 32 | 1 | | 1 | 0 |
| 10 | 2 | 44 | 27 | 1 | | 1 | 2 |
| 10 | 2 | 8 | 32 | 1 | 1 | 1 | 0 |
| 10 | 1 | 60 | 28 | 1 | | 1 | 0 |
| 10 | 2 | 43 | 23 | 1 | | 1 | 0 |
| 10 | 1 | 37 | 29 | 1 | | 1 | 0 |
| 10 | 1 | 24 | 23 | 1 | | 1 | 1 |
| 10 | 2 | 51 | 25 | 1 | | 1 | 1 |
| 10 | 1 | 58 | 28 | 1 | 7 | 1 | 0 |
| 10 | 2 | 59 | 32 | 1 | | 1 | 0 |
| 10 | 2 | 11 | 29 | 1 | 1 | 1 | 1 |
| 10 | 1 | 24 | 32 | 1 | | 1 | 1 |
| 10 | 2 | 40 | 24 | 1 | | 1 | 1 |
| 10 | 1 | 16 | 32 | 1 | 2 | 1 | 1 |
| 10 | 2 | 47 | 20 | 1 | 1 | 1 | 1 |
| 10 | 1 | 18 | 24 | 1 | 1 | 1 | 1 |

| | | | | | | | |
|---|---|---|---|---|---|---|---|
| 10 | 2 | 69 | 19 | 1 | | 1 | 0 |
| 10 | 2 | 39 | 21 | 1 | | 1 | 0 |
| 10 | 1 | 38 | 26 | 1 | | 1 | 0 |
| 10 | 2 | 64 | 31 | 1 | | 1 | 1 |
| 10 | 2 | 41 | 32 | 1 | | 1 | 1 |
| 10 | 1 | 9 | 32 | 1 | | 1 | 1 |
| 10 | 2 | 56 | 21 | 1 | | 1 | 1 |
| 10 | 1 | 48 | 23 | 1 | | 1 | 1 |
| 10 | 2 | 15 | 24 | 1 | | 1 | 1 |
| 10 | 2 | 46 | 22 | 1 | 2 | 1 | 1 |
| 10 | 1 | 54 | 21 | 1 | | 1 | 1 |
| 10 | 1 | 62 | 30 | 1 | | 1 | 1 |
| 10 | 1 | 15 | 30 | 1 | | 1 | 1 |
| 10 | 2 | 31 | 24 | 1 | | 1 | 1 |
| 10 | 1 | 66 | 25 | 1 | 6 | 1 | 0 |
| 10 | 2 | 68 | 27 | 1 | 6 | 1 | 0 |
| 10 | 1 | 59 | 25 | 1 | 2 | 1 | 1 |
| 10 | 2 | 63 | 26 | 1 | 2 | 1 | 1 |
| 10 | 1 | 21 | 23 | 1 | 2 | 1 | 0 |
| 10 | 1 | 70 | 23 | 1 | | 1 | 1 |
| 10 | 2 | 70 | 31 | 1 | | 1 | 1 |
| 10 | 2 | 58 | 23 | 1 | | 1 | 0 |
| 10 | 2 | 73 | 27 | 1 | | 1 | 0 |
| 10 | 2 | 65 | 29 | 1 | -2 | 1 | 1 |
| 10 | 2 | 55 | 22 | 1 | | 1 | 1 |
| 10 | 1 | 13 | 27 | 1 | 8 | 1 | 1 |
| 10 | 1 | 70 | 24 | 1 | | 1 | 2 |
| 10 | 1 | 38 | 24 | 1 | | 1 | 1 |
| 10 | 2 | 38 | 26 | 1 | 1 | 1 | 0 |
| 10 | 1 | 55 | 22 | 1 | 1 | 1 | 1 |
| 10 | 2 | 59 | 21 | 1 | | 1 | 1 |
| 10 | 1 | 46 | 27 | 1 | | 1 | 1 |
| 10 | 2 | 22 | 27 | 1 | | 1 | 1 |
| 10 | 2 | 30 | 31 | 1 | | 1 | 0 |
| 10 | 1 | 48 | 32 | 1 | | 2 | 1 |
| 10 | 2 | 58 | 20 | 1 | | 1 | 1 |
| 10 | 2 | 24 | 28 | 1 | 1 | 1 | 1 |
| 10 | 2 | 35 | 24 | 1 | | 1 | 1 |
| 10 | 1 | 28 | 21 | 1 | | 1 | 1 |
| 10 | 1 | 59 | 19 | 1 | | 1 | 1 |
| 10 | 2 | 60 | 26 | 1 | | 1 | 1 |
| 10 | 1 | 72 | 30 | 1 | 3 | 1 | 0 |
| 10 | 1 | 40 | 25 | 1 | 2 | 1 | 1 |
| 10 | 1 | 10 | 30 | 1 | 1 | 1 | 0 |
| 10 | 2 | 61 | 26 | 1 | 3 | 1 | 0 |
| 10 | 2 | 12 | 21 | 1 | 0 | 1 | 1 |
| 10 | 1 | 27 | 25 | 1 | | 1 | 1 |
| 10 | 1 | 32 | 29 | 1 | | 1 | 0 |
| 10 | 2 | 57 | 23 | 1 | | 1 | 1 |
| 10 | 2 | 56 | 30 | 1 | | 1 | 1 |

| | | | | | | | |
|---|---|---|---|---|---|---|---|
| 10 | 1 | 55 | 31 | 1 | | 1 | 0 |
| 10 | 2 | 19 | 24 | 1 | | 1 | 1 |
| 10 | 1 | 73 | 32 | 1 | | 1 | 1 |
| 10 | 2 | 27 | 26 | 1 | | 1 | 0 |
| 10 | 1 | 50 | 24 | 1 | 4 | 1 | 1 |
| 10 | 2 | 54 | 25 | 1 | 4 | 1 | 1 |
| 10 | 1 | 51 | 21 | 1 | | 1 | 1 |
| 10 | 2 | 44 | 23 | 1 | | 1 | 1 |
| 10 | 2 | 52 | 28 | 1 | | 1 | 1 |
| 10 | 1 | 45 | 20 | 1 | | 1 | 1 |
| 10 | 1 | 57 | 25 | 1 | | 1 | 1 |
| 10 | 1 | 51 | 28 | 1 | | 1 | 1 |
| 10 | 1 | 75 | 31 | 1 | | 1 | 1 |
| 10 | 2 | 17 | 26 | 1 | | 1 | 1 |
| 10 | 2 | 32 | 27 | 1 | | 1 | 1 |
| 10 | 1 | 52 | 29 | 1 | | 1 | 1 |
| 10 | 2 | 59 | 21 | 1 | | 1 | 1 |
| 10 | 2 | 16 | 23 | 1 | 1 | 1 | 1 |
| 10 | 2 | 49 | 25 | 1 | 1 | 1 | 1 |
| 10 | 1 | 43 | 29 | 1 | 1 | 1 | 1 |
| 10 | 1 | 51 | 27 | 1 | 1 | 1 | 1 |
| 10 | 1 | 31 | 27 | 1 | | 1 | 1 |
| 10 | 2 | 61 | 29 | 1 | | 1 | 1 |
| 10 | 2 | 55 | 32 | 1 | 2 | 1 | 1 |
| 10 | 2 | 55 | 24 | 1 | | 1 | 0 |
| 10 | 1 | 76 | 27 | 1 | | 1 | 0 |
| 10 | 2 | 51 | 32 | 1 | | 1 | 0 |
| 10 | 1 | 68 | 18 | 1 | | 1 | 1 |
| 10 | 2 | 37 | 18 | 1 | | 1 | 1 |
| 10 | 2 | 22 | 28 | 1 | | 1 | 1 |
| 10 | 2 | 45 | 22 | 1 | | 1 | 0 |
| 10 | 2 | 58 | 28 | 1 | | 1 | 0 |
| 10 | 2 | 55 | 26 | 1 | | 1 | 1 |
| 10 | 1 | 24 | 25 | 1 | | 1 | 1 |
| 10 | 1 | 35 | 32 | 1 | | 1 | 1 |
| 10 | 2 | 62 | 32 | 1 | | 1 | 2 |
| 10 | 2 | 14 | 26 | 1 | | 1 | 1 |
| 10 | 2 | 35 | 23 | 1 | | 1 | 0 |
| 10 | 2 | 52 | 29 | 1 | | 1 | 0 |
| 10 | 2 | 47 | 20 | 1 | | 1 | 0 |
| 10 | 1 | 34 | 18 | 1 | 1 | 1 | 1 |
| 10 | 2 | 26 | 19 | 1 | 2 | 1 | 1 |
| 10 | 2 | 35 | 28 | 1 | | 1 | 1 |
| 10 | 2 | 30 | 23 | 1 | | 1 | 0 |
| 10 | 1 | 58 | 21 | 1 | | 1 | 1 |
| 10 | 2 | 34 | 27 | 1 | | 1 | 1 |
| 10 | 2 | 63 | 20 | 1 | | 1 | 1 |
| 10 | 1 | 60 | 21 | 1 | | 1 | 1 |
| 10 | 2 | 30 | 26 | 1 | | 1 | 1 |
| 10 | 2 | 50 | 22 | 1 | 2 | 1 | 0 |

| | | | | | | | |
|---|---|---|---|---|---|---|---|
| 10 | 2 | 49 | 30 | 1 | 4 | 1 | 0 |
| 10 | 1 | 47 | 26 | 1 | 5 | 1 | 0 |
| 10 | 2 | 50 | 25 | 1 | 6 | 1 | 1 |
| 10 | 1 | 10 | 22 | 1 | 1 | 1 | 1 |
| 10 | 2 | 61 | 32 | 1 | | 1 | 1 |
| 10 | 1 | 10 | 23 | 1 | | 1 | 1 |
| 10 | 2 | 16 | 22 | 1 | | 1 | 1 |
| 10 | 2 | 14 | 30 | 1 | | 1 | 1 |
| 10 | 1 | 49 | 28 | 1 | 1 | 1 | 1 |
| 10 | 1 | 35 | 25 | 1 | | 1 | 0 |
| 10 | 2 | 29 | 21 | 1 | | 1 | 1 |
| 10 | 1 | 7 | 27 | 1 | | 1 | 0 |
| 10 | 2 | 39 | 23 | 1 | | 1 | 1 |
| 10 | 1 | 84 | 27 | 1 | | 1 | 0 |
| 10 | 2 | 17 | 29 | 1 | | 1 | 1 |
| 10 | 2 | 47 | 31 | 1 | | 1 | 1 |
| 10 | 2 | 28 | 25 | 1 | | 1 | 1 |
| 10 | 2 | 32 | 26 | 1 | | 1 | 1 |
| 10 | 2 | 56 | 29 | 1 | 9 | 1 | 1 |
| 10 | 2 | 60 | 30 | 1 | | 1 | 1 |
| 10 | 1 | 25 | 23 | 1 | | 1 | 0 |
| 10 | 1 | 23 | 31 | 1 | -1 | 1 | 1 |
| 10 | 2 | 32 | 22 | 1 | | 1 | 1 |
| 10 | 2 | 33 | 35 | 1 | 3 | 1 | 1 |
| 10 | 2 | 5 | 30 | 1 | | 1 | 0 |
| 10 | 2 | 38 | 27 | 1 | | 1 | 1 |
| 10 | 2 | 39 | 28 | 1 | | 1 | 1 |
| 10 | 1 | 62 | 20 | 1 | | 1 | 0 |
| 10 | 2 | 28 | 24 | 1 | | 1 | 1 |
| 10 | 1 | 26 | 20 | 1 | | 1 | 1 |
| 10 | 1 | 36 | 28 | 1 | | 1 | 1 |
| 10 | 1 | 61 | 31 | 1 | 0 | 1 | 0 |
| 10 | 2 | 3 | 30 | 1 | | 1 | 1 |
| 10 | 2 | 28 | 32 | 1 | | 1 | 1 |
| 10 | 2 | 64 | 32 | 1 | 2 | 1 | 0 |
| 10 | 2 | 28 | 21 | 1 | | 1 | 1 |
| 10 | 1 | 55 | 26 | 1 | 1 | 1 | 1 |
| 10 | 2 | 21 | 29 | 1 | | 1 | 1 |
| 10 | 1 | 64 | 22 | 1 | 4 | 1 | 1 |
| 10 | 1 | 36 | 23 | 1 | 5 | 1 | 1 |
| 10 | 2 | 65 | 27 | 1 | 8 | 1 | 1 |
| 10 | 2 | 60 | 24 | 1 | | 1 | 1 |
| 10 | 1 | 53 | 26 | 1 | | 1 | 1 |
| 10 | 2 | 51 | 26 | 1 | | 1 | 1 |
| 10 | 2 | 59 | 32 | 1 | | 1 | 1 |
| 10 | 1 | 84 | 22 | 1 | 3 | 1 | 0 |
| 10 | 1 | 48 | 19 | 1 | | 1 | 1 |
| 10 | 2 | 30 | 26 | 1 | | 1 | 0 |
| 10 | 2 | 62 | 24 | 1 | | 1 | 0 |
| 10 | 2 | 26 | 21 | 1 | | 1 | 1 |

| | | | | | | | |
|---|---|---|---|---|---|---|---|
| 10 | 2 | 30 | 23 | 1 | | 1 | 1 |
| 10 | 2 | 25 | 27 | 1 | | 1 | 1 |
| 10 | 2 | 27 | 29 | 1 | | 1 | 1 |
| 10 | 2 | 5 | 29 | 1 | 3 | 1 | 1 |
| 10 | 2 | 69 | 22 | 1 | | 1 | 1 |
| 10 | 1 | 66 | 27 | 1 | | 1 | 1 |
| 10 | 1 | 5 | 29 | 1 | | 1 | 1 |
| 10 | 1 | 21 | 30 | 1 | | 1 | 1 |
| 10 | 2 | 42 | 21 | 1 | | 1 | 0 |
| 10 | 2 | 58 | 22 | 1 | | 1 | 1 |
| 10 | 2 | 7 | 30 | 1 | | 1 | 1 |
| 10 | 1 | 25 | 22 | 1 | 1 | 1 | 1 |
| 10 | 2 | 56 | 20 | 1 | 2 | 1 | 1 |
| 10 | 2 | 45 | 20 | 1 | | 1 | 1 |
| 10 | 1 | 9 | 25 | 1 | | 1 | 1 |
| 10 | 1 | 31 | 26 | 1 | | 1 | 1 |
| 10 | 1 | 53 | 24 | 1 | -2 | 1 | 0 |
| 10 | 1 | 16 | 24 | 1 | 2 | 1 | 0 |
| 10 | 2 | 25 | 28 | 1 | | 1 | 0 |
| 10 | 2 | 77 | 31 | 1 | | 1 | 0 |
| 10 | 1 | 70 | 31 | 1 | | 1 | 0 |
| 10 | 2 | 27 | 20 | 1 | | 1 | 1 |
| 10 | 1 | 24 | 28 | 1 | | 1 | 0 |
| 10 | 1 | 26 | 26 | 1 | 1 | 1 | 1 |
| 10 | 1 | 39 | 22 | 1 | | 1 | 1 |
| 10 | 2 | 25 | 29 | 1 | 1 | 1 | 1 |
| 10 | 1 | 22 | 20 | 1 | | 1 | 1 |
| 10 | 2 | 48 | 22 | 1 | | 1 | 1 |
| 10 | 2 | 32 | 24 | 1 | | 1 | 1 |
| 10 | 2 | 6 | 22 | 1 | | 1 | 1 |
| 10 | 2 | 57 | 24 | 1 | | 1 | 1 |
| 10 | 2 | 81 | 20 | 1 | | 1 | 1 |
| 10 | 2 | 20 | 26 | 1 | 7 | 1 | 1 |
| 10 | 1 | 47 | 24 | 1 | | 1 | 0 |
| 10 | 1 | 11 | 28 | 1 | | 1 | 0 |
| 10 | 1 | 16 | 30 | 1 | | 1 | 0 |
| 10 | 2 | 50 | 30 | 1 | | 1 | 0 |
| 10 | 1 | 71 | 24 | 1 | | 1 | 1 |
| 10 | 1 | 39 | 25 | 1 | | 1 | 1 |
| 10 | 2 | 62 | 25 | 1 | | 1 | 1 |
| 10 | 2 | 22 | 25 | 1 | | 1 | 1 |
| 10 | 2 | 56 | 26 | 1 | | 1 | 1 |
| 10 | 2 | 63 | 19 | 1 | | 1 | 0 |
| 10 | 2 | 65 | 18 | 1 | | 1 | 1 |
| 10 | 1 | 24 | 21 | 1 | | 1 | 1 |
| 10 | 2 | 32 | 29 | 1 | | 1 | 1 |
| 10 | 1 | 62 | 31 | 1 | | 1 | 1 |
| 10 | 1 | 59 | 31 | 1 | | 1 | 1 |
| 10 | 2 | 58 | 30 | 1 | | 1 | 0 |
| 10 | 1 | 51 | 23 | 1 | | 1 | 1 |

| | | | | | | | |
|---|---|---|---|---|---|---|---|
| 10 | 1 | 58 | 29 | 1 | | 1 | 0 |
| 10 | 1 | 81 | 23 | 1 | | 1 | 1 |
| 10 | 1 | 20 | 19 | 1 | | 1 | 1 |
| 10 | 2 | 58 | 28 | 1 | | 1 | 1 |
| 10 | 1 | 53 | 23 | 1 | 2 | 1 | 1 |
| 10 | 1 | 20 | 19 | 1 | | 1 | 0 |
| 10 | 1 | 23 | 20 | 1 | | 1 | 0 |
| 10 | 2 | 53 | 20 | 1 | 1 | 1 | 1 |
| 10 | 2 | 74 | 21 | 1 | 1 | 1 | 1 |
| 10 | 1 | 31 | 25 | 1 | | 1 | 1 |
| 10 | 2 | 63 | 26 | 1 | | 1 | 0 |
| 10 | 2 | 61 | 22 | 1 | | 1 | 1 |
| 10 | 1 | 57 | 22 | 1 | | 1 | 1 |
| 10 | 2 | 31 | 26 | 1 | 1 | 1 | 0 |
| 10 | 1 | 54 | 32 | 1 | | 1 | 0 |
| 10 | 1 | 57 | 24 | 1 | 1 | 1 | 1 |
| 10 | 2 | 25 | 28 | 1 | 1 | 1 | 1 |
| 10 | 2 | 53 | 30 | 1 | 2 | 1 | 1 |
| 10 | 1 | 34 | 20 | 1 | 2 | 1 | 1 |
| 10 | 2 | 40 | 29 | 1 | 4 | 1 | 1 |
| 10 | 1 | 8 | 31 | 1 | 4 | 1 | 1 |
| 10 | 1 | 68 | 22 | 1 | | 1 | 1 |
| 10 | 2 | 25 | 27 | 1 | | 2 | 1 |
| 10 | 1 | 40 | 26 | 1 | 7 | 1 | 1 |
| 10 | 1 | 30 | 31 | 1 | 1 | 1 | 0 |
| 10 | 2 | 89 | 23 | 1 | | 2 | 0 |
| 10 | 2 | 43 | 17 | 1 | | 1 | 1 |
| 10 | 2 | 64 | 27 | 1 | | 1 | 1 |
| 10 | 1 | 64 | 31 | 1 | | 1 | 1 |
| 10 | 1 | 20 | 26 | 1 | 2 | 1 | 1 |
| 10 | 1 | 4 | 29 | 1 | 1 | 1 | 1 |
| 10 | 1 | 38 | 24 | 1 | 3 | 1 | 1 |
| 10 | 2 | 38 | 20 | 1 | | 1 | 1 |
| 10 | 2 | 38 | 21 | 1 | | 1 | 1 |
| 10 | 1 | 83 | 28 | 1 | | 1 | 1 |
| 10 | 1 | 21 | 30 | 1 | | 1 | 1 |
| 10 | 1 | 80 | 31 | 1 | | 1 | 1 |
| 10 | 2 | 21 | 25 | 1 | 1 | 1 | 1 |
| 10 | 1 | 56 | 32 | 1 | 1 | 1 | 1 |
| 10 | 2 | 20 | 23 | 1 | | 1 | 1 |
| 10 | 2 | 24 | 30 | 1 | | 1 | 1 |
| 10 | 2 | 16 | 23 | 1 | | 1 | 1 |
| 10 | 2 | 9 | 29 | 1 | | 1 | 1 |
| 10 | 1 | 55 | 22 | 1 | 1 | 1 | 1 |
| 10 | 2 | 62 | 22 | 1 | 0 | 1 | 1 |
| 10 | 2 | 46 | 24 | 1 | 2 | 1 | 1 |
| 10 | 2 | 52 | 26 | 1 | 3 | 1 | 1 |
| 10 | 2 | 26 | 31 | 1 | | 1 | 1 |
| 10 | 1 | 54 | 29 | 1 | 1 | 1 | 0 |
| 10 | 2 | 56 | 20 | 1 | | 1 | 0 |

| | | | | | | | |
|---|---|---|---|---|---|---|---|
| 10 | 1 | 58 | 21 | 1 | 2 | 1 | 1 |
| 10 | 2 | 3 | 29 | 1 | 1 | 1 | 1 |
| 10 | 1 | 51 | 26 | 1 | | 1 | 1 |
| 10 | 2 | 5 | 27 | 1 | | 1 | 1 |
| 10 | 1 | 44 | 21 | 1 | 2 | 1 | 1 |
| 10 | 2 | 84 | 19 | 1 | 2 | 1 | 1 |
| 10 | 2 | 84 | 21 | 1 | 2 | 1 | 1 |
| 10 | 1 | 52 | 18 | 1 | | 1 | 1 |
| 10 | 2 | 29 | 22 | 1 | | 1 | 1 |
| 10 | 1 | 39 | 27 | 1 | | 1 | 1 |
| 10 | 1 | 48 | 25 | 1 | 2 | 1 | 1 |
| 10 | 2 | 51 | 30 | 1 | | 1 | 1 |
| 10 | 2 | 50 | 30 | 1 | | 1 | 1 |
| 10 | 1 | 55 | 32 | 1 | | 1 | 1 |
| 10 | 2 | 25 | 24 | 1 | 3 | 1 | 0 |
| 10 | 1 | 36 | 22 | 1 | | 1 | 0 |
| 10 | 2 | 36 | 23 | 1 | | 1 | 0 |
| 10 | 1 | 63 | 26 | 1 | | 1 | 0 |
| 10 | 1 | 49 | 29 | 1 | | 1 | 0 |
| 10 | 1 | 22 | 30 | 1 | | 1 | 0 |
| 10 | 1 | 73 | 31 | 1 | | 1 | 0 |
| 10 | 1 | 63 | 29 | 1 | | 1 | 1 |
| 10 | 2 | 29 | 27 | 1 | | 1 | 1 |
| 10 | 2 | 62 | 29 | 1 | | 1 | 1 |
| 10 | 2 | 50 | 25 | 1 | | 1 | 1 |
| 10 | 1 | 46 | 25 | 1 | | 1 | 1 |
| 10 | 2 | 53 | 30 | 1 | | 1 | 1 |
| 10 | 1 | 41 | 23 | 1 | 3 | 1 | 1 |
| 10 | 2 | 51 | 21 | 1 | | 1 | 1 |
| 10 | 1 | 70 | 26 | 1 | 2 | 1 | 1 |
| 10 | 2 | 18 | 26 | 1 | | 1 | 1 |
| 10 | 1 | 55 | 28 | 1 | | 1 | 1 |
| 10 | 1 | 53 | 27 | 1 | | 1 | 1 |
| 10 | 2 | 57 | 21 | 1 | | 1 | 1 |
| 10 | 1 | 63 | 22 | 1 | | 1 | 1 |
| 10 | 1 | 24 | 24 | 1 | | 1 | 1 |
| 10 | 1 | 23 | 32 | 1 | | 1 | 1 |
| 10 | 1 | 50 | 32 | 1 | | 1 | 1 |
| 10 | 1 | 4 | 23 | 1 | | 1 | 1 |
| 10 | 2 | 57 | 20 | 1 | | 1 | 1 |
| 10 | 1 | 40 | 19 | 1 | | 1 | 1 |
| 10 | 2 | 52 | 26 | 1 | | 1 | 1 |
| 10 | 2 | 50 | 20 | 1 | | 1 | 1 |
| 10 | 1 | 44 | 21 | 1 | | 1 | 1 |
| 10 | 2 | 49 | 28 | 1 | | 1 | 1 |
| 10 | 2 | 32 | 32 | 1 | | 1 | 0 |
| 10 | 1 | 13 | 31 | 1 | | 1 | 1 |
| 10 | 1 | 80 | 23 | 1 | | 1 | 1 |
| 10 | 2 | 71 | 23 | 1 | | 1 | 1 |
| 10 | 1 | 31 | 21 | 1 | | 1 | 1 |

| | | | | | | | |
|---|---|---|---|---|---|---|---|
| 10 | 2 | 57 | 21 | 1 | | 1 | 1 |
| 10 | 1 | 64 | 19 | 1 | | 1 | 0 |
| 10 | 1 | 37 | 21 | 1 | | 1 | 0 |
| 10 | 2 | 40 | 27 | 1 | | 1 | 0 |
| 10 | 2 | 40 | 28 | 1 | 2 | 1 | 1 |
| 10 | 2 | 33 | 21 | 1 | 1 | 1 | 1 |
| 10 | 2 | 41 | 27 | 1 | | 1 | 1 |
| 10 | 1 | 48 | 20 | 1 | | 1 | 0 |
| 10 | 2 | 9 | 25 | 1 | | 1 | 0 |
| 10 | 2 | 27 | 28 | 1 | | 1 | 0 |
| 10 | 1 | 20 | 29 | 1 | | 1 | 0 |
| 10 | 1 | 56 | 23 | 1 | | 1 | 0 |
| 10 | 2 | 40 | 20 | 1 | | 1 | 1 |
| 10 | 1 | 23 | 27 | 1 | | 1 | 1 |
| 10 | 2 | 55 | 20 | 1 | | 1 | 1 |
| 10 | 2 | 61 | 25 | 1 | | 1 | 1 |
| 10 | 1 | 31 | 25 | 1 | | 1 | 1 |
| 10 | 2 | 8 | 29 | 1 | | 1 | 1 |
| 10 | 1 | 36 | 24 | 1 | | 1 | 1 |
| 10 | 2 | 55 | 31 | 1 | | 1 | 1 |
| 10 | 1 | 3 | 25 | 1 | -4 | 1 | 0 |
| 10 | 2 | 8 | 20 | 1 | | 1 | 0 |
| 10 | 1 | 35 | 24 | 1 | | 1 | 0 |
| 10 | 2 | 33 | 27 | 1 | | 2 | 1 |
| 11 | 2 | 56 | 29 | 1 | 1 | 1 | 1 |
| 11 | 2 | 42 | 23 | 1 | | 1 | 1 |
| 11 | 1 | 60 | 28 | 1 | | 1 | 0 |
| 11 | 1 | 8 | 33 | 1 | | 1 | 0 |
| 11 | 1 | 82 | 30 | 1 | | 2 | 1 |
| 11 | 2 | 44 | 28 | 1 | 4 | 1 | 0 |
| 11 | 2 | 62 | 18 | 1 | | 1 | 1 |
| 11 | 1 | 61 | 20 | 1 | | 1 | 1 |
| 11 | 2 | 41 | 22 | 1 | | 1 | 1 |
| 11 | 2 | 80 | 22 | 1 | | 1 | 1 |
| 11 | 1 | 57 | 25 | 1 | 1 | 1 | 1 |
| 11 | 1 | 60 | 24 | 1 | 3 | 1 | 0 |
| 11 | 1 | 46 | 23 | 1 | | 1 | 0 |
| 11 | 2 | 31 | 24 | 1 | 0 | 1 | 1 |
| 11 | 2 | 58 | 26 | 1 | 2 | 1 | 1 |
| 11 | 2 | 19 | 23 | 1 | | 1 | 1 |
| 11 | 1 | 30 | 26 | 1 | | 1 | 1 |
| 11 | 2 | 3 | 30 | 1 | -1 | 1 | 2 |
| 11 | 1 | 56 | 24 | 1 | 5 | 1 | 2 |
| 11 | 1 | 21 | 22 | 1 | | 1 | 1 |
| 11 | 1 | 19 | 23 | 1 | | 1 | 1 |
| 11 | 2 | 51 | 24 | 1 | | 1 | 1 |
| 11 | 1 | 62 | 27 | 1 | | 1 | 1 |
| 11 | 2 | 3 | 25 | 1 | | 1 | 1 |
| 11 | 2 | 7 | 30 | 1 | | 1 | 1 |
| 11 | 2 | 51 | 29 | 1 | | 1 | 1 |

| | | | | | | | |
|---|---|---|---|---|---|---|---|
| 11 | 1 | 55 | 27 | 1 | | 1 | 1 |
| 11 | 1 | 33 | 28 | 1 | | 1 | 0 |
| 11 | 1 | 6 | 23 | 1 | -1 | 1 | 1 |
| 11 | 2 | 4 | 18 | 1 | | 1 | 1 |
| 11 | 2 | 9 | 29 | 1 | | 1 | 0 |
| 11 | 1 | 40 | 24 | 1 | 1 | 1 | 1 |
| 11 | 2 | 10 | 22 | 1 | | 1 | 1 |
| 11 | 2 | 7 | 24 | 1 | | 1 | 1 |
| 11 | 1 | 34 | 25 | 1 | | 1 | 1 |
| 11 | 2 | 34 | 27 | 1 | | 1 | 1 |
| 11 | 2 | 66 | 22 | 1 | 2 | 1 | 1 |
| 11 | 1 | 86 | 22 | 1 | | 1 | 1 |
| 11 | 2 | 45 | 24 | 1 | | 1 | 1 |
| 11 | 2 | 56 | 24 | 1 | 3 | 1 | 0 |
| 11 | 1 | 37 | 24 | 1 | | 1 | 0 |
| 11 | 1 | 36 | 26 | 1 | | 1 | 0 |
| 11 | 1 | 43 | 23 | 1 | 3 | 1 | 1 |
| 11 | 2 | 11 | 25 | 1 | 3 | 1 | 1 |
| 11 | 1 | 16 | 25 | 1 | 4 | 1 | 1 |
| 11 | 1 | 14 | 22 | 1 | 5 | 1 | 1 |
| 11 | 2 | 2 | 23 | 1 | | 1 | 1 |
| 11 | 1 | 19 | 29 | 1 | | 1 | 1 |
| 11 | 2 | 83 | 28 | 1 | | 2 | 1 |
| 11 | 2 | 30 | 24 | 1 | 3 | 1 | 0 |
| 11 | 2 | 13 | 17 | 1 | | 1 | 1 |
| 11 | 1 | 45 | 17 | 1 | 1 | 1 | 1 |
| 11 | 2 | 54 | 23 | 1 | | 1 | 1 |
| 11 | 1 | 25 | 30 | 1 | | 1 | 1 |
| 11 | 1 | 52 | 23 | 1 | | 1 | 1 |
| 11 | 2 | 54 | 28 | 1 | | 1 | 1 |
| 11 | 1 | 67 | 28 | 1 | | 1 | 0 |
| 11 | 2 | 40 | 22 | 1 | | 1 | 1 |
| 11 | 1 | 33 | 22 | 1 | | 1 | 1 |
| 11 | 2 | 23 | 23 | 1 | | 1 | 1 |
| 11 | 2 | 38 | 25 | 1 | | 1 | 1 |
| 11 | 2 | 20 | 26 | 1 | | 1 | 1 |
| 11 | 1 | 47 | 30 | 1 | | 1 | 1 |
| 11 | 2 | 17 | 30 | 1 | | 1 | 1 |
| 11 | 1 | 34 | 25 | 1 | 1 | 1 | 2 |
| 11 | 2 | 52 | 26 | 1 | | 1 | 0 |
| 11 | 2 | 39 | 30 | 1 | | 1 | 0 |
| 11 | 1 | 31 | 27 | 1 | | 1 | 0 |
| 11 | 1 | 4 | 23 | 1 | | 1 | 1 |
| 11 | 2 | 20 | 28 | 1 | 1 | 1 | 1 |
| 11 | 2 | 59 | 19 | 1 | | 1 | 0 |
| 11 | 1 | 58 | 21 | 1 | 3 | 1 | 1 |
| 11 | 2 | 33 | 20 | 1 | | 1 | 1 |
| 11 | 1 | 70 | 18 | 1 | | 1 | 0 |
| 11 | 2 | 32 | 21 | 1 | | 1 | 0 |
| 11 | 1 | 47 | 26 | 1 | 1 | 1 | 0 |

| | | | | | | | |
|---|---|---|---|---|---|---|---|
| 11 | 1 | 90 | 22 | 1 | | 2 | 1 |
| 11 | 2 | 30 | 22 | 1 | | 1 | 1 |
| 11 | 1 | 56 | 23 | 1 | | 1 | 1 |
| 11 | 2 | 57 | 28 | 1 | | 2 | 1 |
| 11 | 2 | 51 | 18 | 1 | | 1 | 1 |
| 11 | 1 | 48 | 22 | 1 | | 1 | 1 |
| 11 | 2 | 47 | 25 | 1 | 2 | 1 | 1 |
| 11 | 2 | 69 | 22 | 1 | 4 | 1 | 0 |
| 11 | 1 | 30 | 20 | 1 | 2 | 1 | 1 |
| 11 | 1 | 36 | 20 | 1 | | 1 | 1 |
| 11 | 1 | 45 | 24 | 1 | | 1 | 1 |
| 11 | 2 | 57 | 26 | 1 | | 1 | 1 |
| 11 | 1 | 51 | 23 | 1 | | 1 | 1 |
| 11 | 1 | 28 | 22 | 1 | | 1 | 0 |
| 11 | 1 | 68 | 18 | 1 | 3 | 1 | 1 |
| 11 | 2 | 55 | 22 | 1 | | 1 | 1 |
| 11 | 1 | 36 | 29 | 1 | | 1 | 1 |
| 11 | 1 | 44 | 22 | 1 | 1 | 1 | 1 |
| 11 | 1 | 42 | 26 | 1 | 1 | 1 | 1 |
| 11 | 1 | 58 | 20 | 1 | 2 | 1 | 1 |
| 11 | 2 | 49 | 20 | 1 | | 1 | 1 |
| 11 | 2 | 8 | 22 | 1 | | 1 | 1 |
| 11 | 2 | 44 | 22 | 1 | | 1 | 1 |
| 11 | 1 | 6 | 30 | 1 | | 1 | 1 |
| 11 | 2 | 38 | 30 | 1 | | 1 | 1 |
| 11 | 2 | 22 | 20 | 1 | | 1 | 1 |
| 11 | 2 | 42 | 21 | 1 | | 1 | 1 |
| 11 | 2 | 6 | 24 | 1 | | 1 | 1 |
| 11 | 1 | 39 | 25 | 1 | | 1 | 1 |
| 11 | 1 | 22 | 22 | 1 | | 1 | 1 |
| 11 | 1 | 48 | 25 | 1 | | 1 | 0 |
| 11 | 2 | 58 | 22 | 1 | | 1 | 1 |
| 11 | 1 | 57 | 22 | 1 | | 1 | 1 |
